# Stage-specific function of sphingolipid synthases in African trypanosomes

**DOI:** 10.1128/mbio.03501-24

**Published:** 2024-12-16

**Authors:** Norton Heise, Carolina M. Koeller, Mohamed Sharif, James D. Bangs

**Affiliations:** 1 Instituto de Biofísica Carlos Chagas Filho, Universidade Federal do Rio de Janeiro, Rio de Janeiro, Brazil; 2 Department of Microbiology & Immunology, Jacobs School of Medicine and Biomedical Sciences, University at Buffalo, Buffalo, New York, USA; Stanford University, Stanford, California, USA

**Keywords:** trypanosome, sphingolipid, sphingolipid synthase, sphingomyelin, inositolphosphorylceramide

## Abstract

The protozoan parasite *Trypanosoma brucei* is the only known eukaryote capable of synthesizing the three main phosphosphingolipids: sphingomyelin (SM), inositol phosphorylceramide (IPC), and ethanolamine phosphorylceramide (EPC). It has four paralogous genes encoding sphingolipid synthases (*TbSLS1–4*). TbSLS1 is a dedicated IPC synthase, TbSLS2 is a dedicated EPC synthase, and TbSLS3 and TbSLS4 are bifunctional SM/EPC synthases. IPC synthesis occurs exclusively in the procyclic insect stage (PCF), EPC is limited to the mammalian bloodstream form (BSF), and SM is synthesized throughout the life cycle. TbSLSs are indispensable for the viability of BSF and are, thus, potential drug targets. The relative stage-specific expression of each *TbSLS* paralog was compared, and the results match phosphosphingolipid content. Induction of pan-specific RNAi silencing was lethal in both BSF and PCF. To investigate individual TbSLS functions, separate HA-tagged genes, recoded to be RNAi-resistant (RNAi^R^), were engineered to replace a single allele of the entire *TbSLS* locus within parental BSF and PCF RNAi cell lines. RNAi^R^ *TbSLS3* and *TbSLS4* both rescued BSF growth under silencing. Expression of RNAi^R^ *TbSLS1*, normally repressed in BSF, did not rescue BSF viability but was not detrimental to normal *in vitro* growth. RNAi^R^ *TbSLS1*, *TbSLS3*, and *TbSLS4* were each sufficient to rescue PCF growth, indicating IPC is not essential for PCF viability *in vitro*. All TbSLSs localize to distal Golgi compartments in both BSF and PCF cells. These findings raise interesting questions about the roles of individual phosphosphingolipids in *in vivo* infection of the mammalian and tsetse hosts.

**IMPORTANCE:**

African trypanosomes are eukaryotic pathogens that cause human and veterinary African trypanosomaisis. Uniquely, they synthesize all three major phosphosphingolipid species using four distinct sphingolipid synthases (SLS). This work details the function of each SLS in both bloodstream and insect form parasites. Novel and unexpected sphingolipid dependences are found in each stage. These results are consistent with this metabolic pathway being a valid target for chemotherapeutic intervention.

## INTRODUCTION

Phosphosphingolipids (PSLs) are essential membrane components in all eukaryotic cells, contributing to the stability and fluidity of biological membranes, participating in various cell signaling pathways that regulate cell growth and differentiation, and acting as bioactive lipids that promote cell survival and proliferation (1–4). In addition, PSLs can serve as precursors for the synthesis of phosphatidylcholine and phosphatidylethanolamine, as well as ether lipids and plasmalogens. Sphingolipid synthesis begins with the generation of ceramide in the ER (5, 6). Ceramide is then transported to the Golgi where specific sphingolipid synthases (SLSs) transfer polar head groups from phosphoglycerolipid donors to ceramide acceptors to generate the common phosphosphingolipids (PSLs) (7): sphingomyelin (SM), ethanolamine phosphorylceramide (EPC), and inositol phosphorylceramide (IPC). Different clades of eukaryotes utilize distinct PSLs—SM dominates in vertebrates, EPC in insects, and IPC in plants and in single cell organisms such as protozoa and fungi (8–10).

The Kinetoplastida are an anciently derived group of mostly parasitic protozoa (11). These include the causative agents of Leishmaniasis (*Leishmania* spp.), Chagas disease (*Trypanosoma cruzi*), and African Sleeping Sickness (*T. brucei* spp.) in humans. Compositional analyses indicate the presence of only IPC in *Leishmania* and *T. cruzi* (12, 13), while *T. brucei* contains IPC, SM, and lesser amounts of EPC (14). Interestingly, in *T. brucei,* IPC is only found in the procyclic insect form (PCF) indicating stage-specific regulation of its synthesis (14, 15). SM is found in both PCF and the mammalian bloodstream form (BSF), while EPC is only found in BSF cells, which may simply be an issue of detection sensitivity.

We and others have used homology approaches to identify *SLS* genes in *T. brucei*, *T. cruzi,* and *L. major* (14, 16). Both *T. cruzi* and *Leishmania* have single syntenic *SLS* genes, which by inference from the PSL compositional studies must encode IPC synthases. This was confirmed for *L. major* (*LmjSLS*) by trans-complementation in mammalian cells (16). In contrast, *T. brucei* has a syntenic array of four *SLS* genes (*TbSLS1-4*) (Fig. 2A). Using an *in vitro* translation (IVT) system for the production of polytopic membrane proteins (17), we determined the enzymatic specificity of each in *in vitro* PSL synthesis assays (18, 19). TbSLS1 is a dedicated IPC synthase. This in conjunction with the fact that it is upregulated during differentiation of BSF cells to the PCF stage (20) accounts for the observed stage-specific production of IPC in trypanosomes. TbSLS2 is a dedicated EPC synthase, while TbSLS3 and TbSLS4 are bifunctional SM/EPC synthases. A single residue in lumenal loop III (TbSLS2, N170; TbSLS4, A170) modulates enzymatic specificity, presumably by restricting (TbSLS2) or accommodating (TbSLS4) the larger choline head group required for SM synthesis (18). This work also confirmed that the *T. cruzi* gene (*TcrSLS*) and *LmjSLS* both encode dedicated IPC synthases.

SLSs are part of the larger group of lipid phosphate phosphatases (21) and are predicted to be six trans-helical membrane proteins with the active site on the lumenal face of the Golgi membrane (14, 22, 23). The four TbSLSs are highly similar (>90% identical) with most amino acid sequence diversity found in the cytoplasmic C-terminus (14). However, a single “signature” residue located next to a catalytic histidine in lumenal Loop V is predictive for enzymatic specificity (Ser252 in TbSLS1; Phe252 in TbSLS2-4), and the equivalent residue in LmjSLS and TcrSLS is also a serine (18, 19). The predictive value of this residue was confirmed in the IVT system by site-specific mutagenesis of TbSLS1 and TbSLS3. More recently, we have surveyed the other available kinetoplastid genomes, including the most phylogenetically distant, *Bodo saltans* (24). All have single syntenic SLS orthologs with serine as the signature residue, except *T. vivax*, which has a tyrosine. Again using the IVT system, we confirmed that BsSLS is a *bona fide* IPC synthase, but TvSLS is a bifunctional SM/EPC synthase. Based on these results, we presume that all other kinetoplastid orthologs with a signature serine residue are IPC synthases.

**Fig 1:**
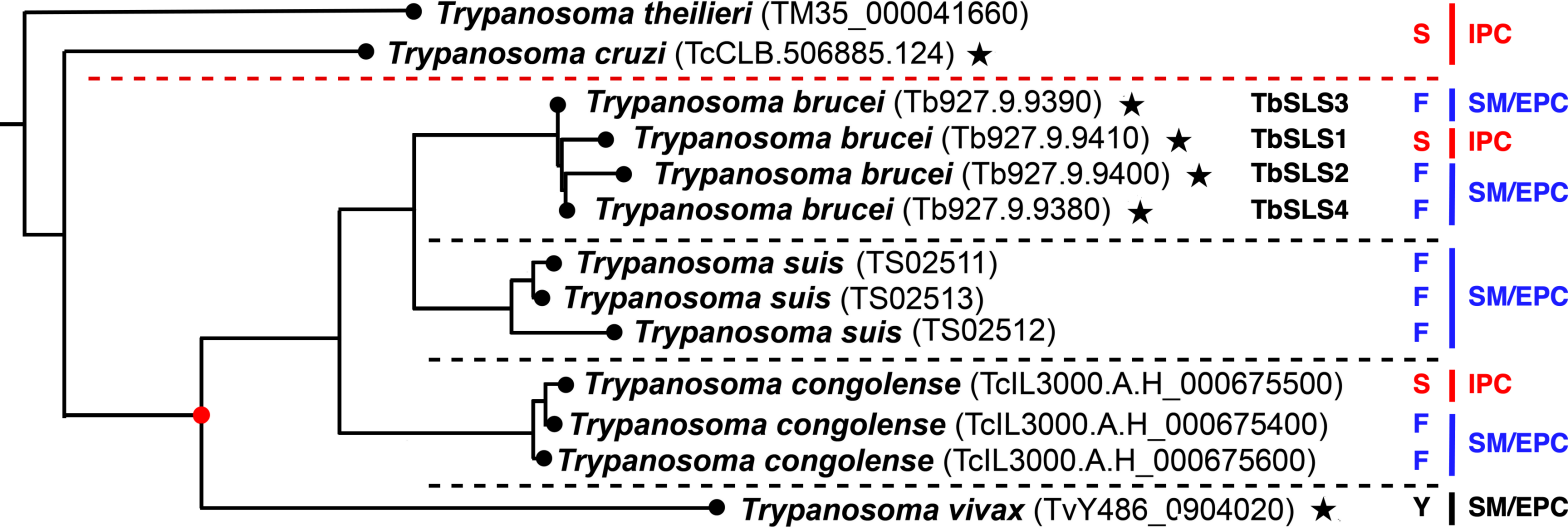
Phylogenetic analysis of SLS sequences in *Trypanosoma*. A maximum likelihood phylogenetic tree of SLS protein sequences across the *Trypanosoma* (TriTrypDB identifers shown in brackets) is presented [adapted from Ciganda et al. (24)]. SLS proteins for which the product specificity (IPC vs SM/EPC) have been empirically determined are indicated with a star. The enzymatic specificity-defining residue for each SLS (serine, red; phenylalanine, blue; tyrosine, black) is indicated in single letter code on the right. The *Stercoraria* and *Salivaria* are separated by a dashed red line. The four Salivarian clades are separated by dashed black lines. The red dot indicates the position of a putative IPC synthase-containing ancestor of the *Salivaria*.

Of all the Kinetoplastida, including *T. vivax,* the earliest diverging of the African (salivarian) trypanosomes, only the later diverging salivarians *T. congolense* (three genes), *T. brucei* (four genes), and *T. suis* (three genes) have amplified the SLS locus (Fig. 1). In doing so, both *T. congolense* and *T. brucei* have maintained one *SLS* gene encoding proteins with proven (*T. brucei*) or predicted (*T. congolense*) IPC synthase activity (24). However, *T. suis*, which is closely related to *T. brucei*, has not retained an IPC synthase—all three genes are predicted SM/EPC synthases. This apparently makes *T. vivax* and *T. suis* the only kinetoplastids that do not require IPC at some stage of their life cycles. The evolutionary aspects of this situation are discussed below and in reference 24.

The diversification of SLS activities in *T. brucei* and *T. congolense* also raises interesting questions concerning the roles of the individual SLSs in these parasites. In the current study, we perform detailed stage-specific analyses of the four TbSLSs, including relative expression levels, ability to sustain *in vitro* viability, and localization. Our findings elucidate hitherto unsuspected dependencies and novel regulatory mechanisms in sphingolipid metabolism in *T. brucei* and highlight additional questions concerning the role(s) of PSLs in natural *in vivo* infections of the mammalian host and tsetse fly vector.

## RESULTS

### Stage-specific expression of TbSLSs

**Fig 2:**
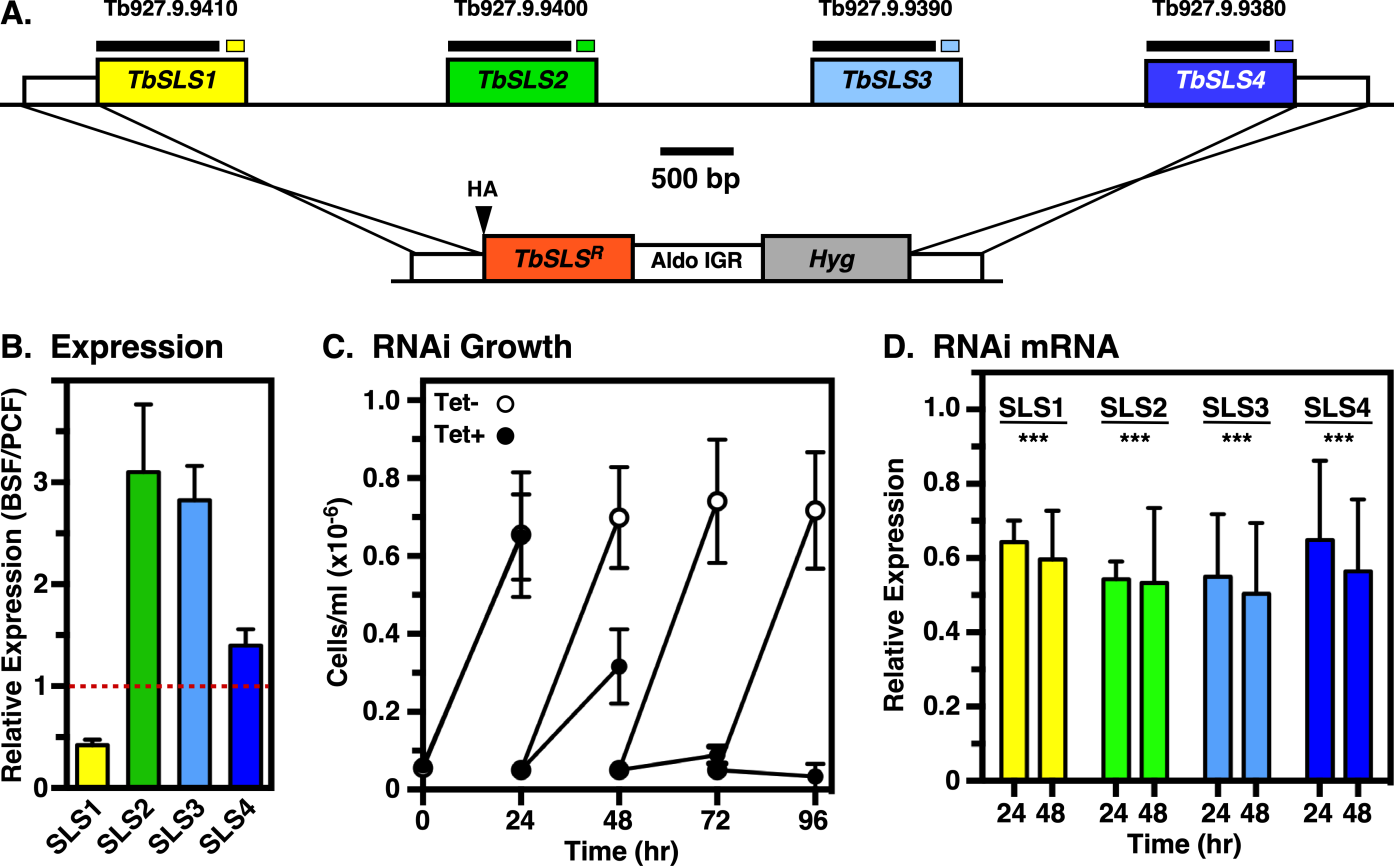
Silencing of native TbSLS in BSF trypanosomes. (A) (Top) Diagram of the *TbSLS* locus. TriTryp database accession numbers are indicated. Black overbars indicate the region of each of targeted by pan-specific RNAi silencing and recoded in the RNAi-resistant gene (Fig. S2 A through D). Colored overbars indicate the approximate position and size of the paralog-specific qRT-PCR target regions for the native genes. (Bottom) Cassette for replacing one *TbSLS* allele with individual RNAi-resistant *TbSLS* genes. The position of the N-terminal HA-tags is indicated. (**B)** The relative stage-specific expression levels of each paralog in parental cell lines were determined by qRT-PCR with primer pairs II-III (see Fig. S1; Table S1) and are expressed as the ratio of BSF to PCF. Red line indicates equal expression. Relative expression between paralogs within either stage cannot be inferred from these data. (**C)** The parental BSF *TbSLS* RNAi cell line with intact *TbSLS* alleles was cultured with and without tetracycline as indicated to induce pan-specific silencing. Cultures were enumerated by manual counting and adjusted to starting density (5 × 10^4^/mL) every 24 h. (**D)** Specific mRNA levels of the individual wild type *TbSLS* paralogs were determined at 24 and 48 h of silencing by qRT-PCR with primer pairs II-III. Data are presented as the ratio of silenced (Tet+) to control (Tet−). Significance relative to control unsilenced cells calculated by one-way Anova: ***, *P* < 0.001. (**B through D)** All data are expressed as mean ± std. dev.

Our previous pan-specific northern analysis of total *TbSLS* mRNA indicated that total expression was ~2-fold higher in PCF vs BSF stages (14). We have now used unique primer sets (Fig. S1, II-III; Table S1) to determine the relative stage-specific expression of each paralog by qRT-PCR. Consistent with the lack of detectable steady-state IPC (14) and its synthesis (see Fig. 4B) in BSF cells, the relative level of *TbSLS1* expression is less than half that of PCF trypanosomes (Fig. 2B), where IPC is the major SL (see Fig. 8B). It is curious that there is a small but real level of TbSLS1 mRNA in BSF cells, yet IPC synthesis is undetectable. Perhaps, there is some additional level of regulation at the translational or post-translational levels that controls this activity. Conversely, levels of *TbSLS2* and *TbSLS3* mRNA were threefold higher, and *TbSLS4* was modestly higher in BSF cells, consistent with the predominance of SM and EPC in this stage.

### Conditional TbSLS expression in BSF trypanosomes

**Fig 3:**
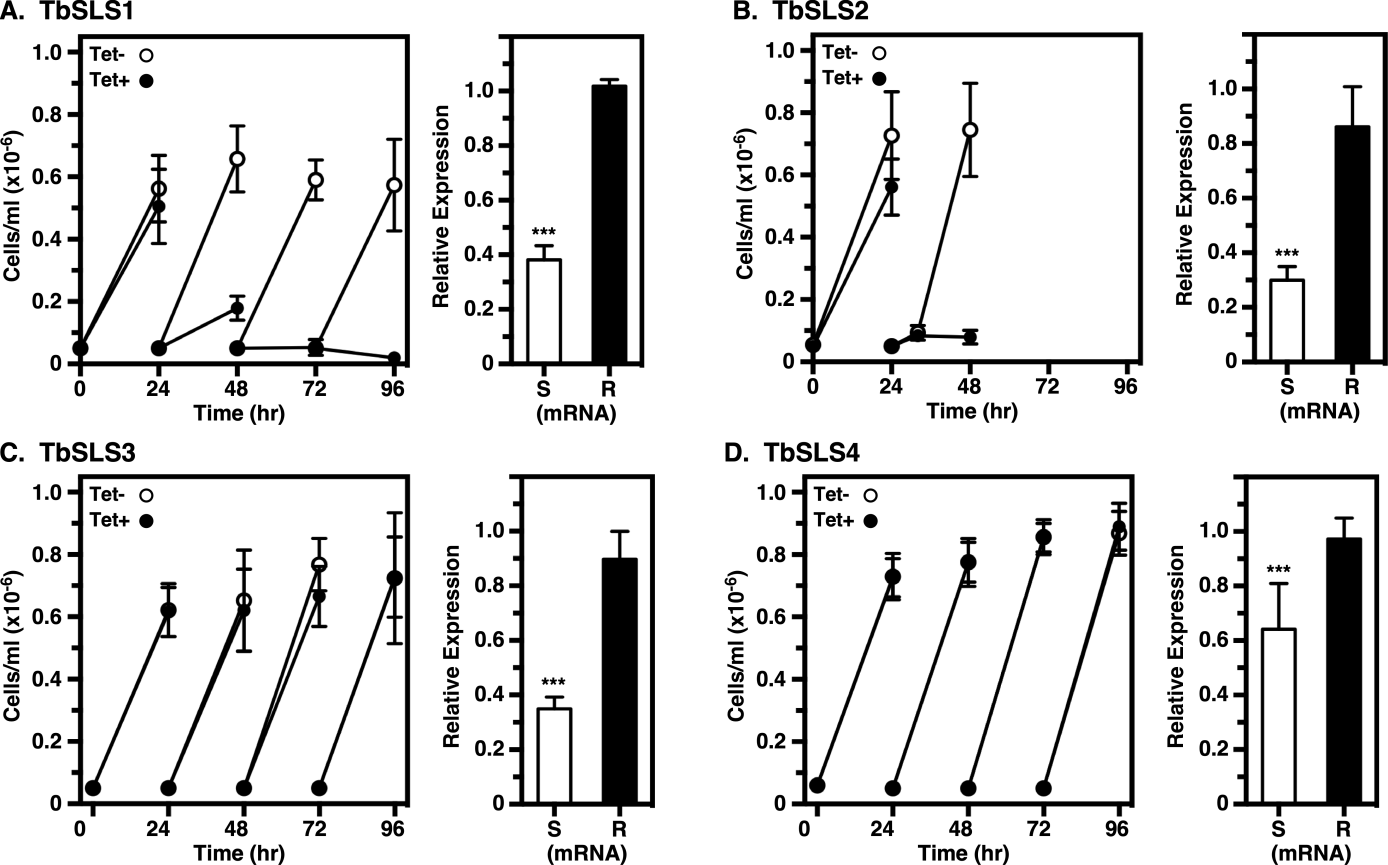
Rescue of BSF RNAi cells by RNAi^R^ TbSLS genes. BSF *TbSLS* RNAi cell lines containing RNAi-resistant *TbSLS1* (**A**), *TbSLS2* (**B**), *TbSLS3* (**C**), or *TbSLS4* (**D**) genes were cultured with and without tetracycline as indicated to induce pan-specific silencing of the native SLS alleles. Cell growth (left panels) was monitored as in Fig. 2C and expression levels (right panels) of the native (**S**) and RNAi-resistant (**R**) genes were monitored by qRT-PCR at 24 h with primer pairs I^S^-II and I^R^-II, respectively (see Fig. S1). (**A through D)** All data are expressed as mean ± std. dev. Significance relative to control unsilenced cells calculated by one-way Anova: ***, *P* < 0.001.

**Fig 4:**
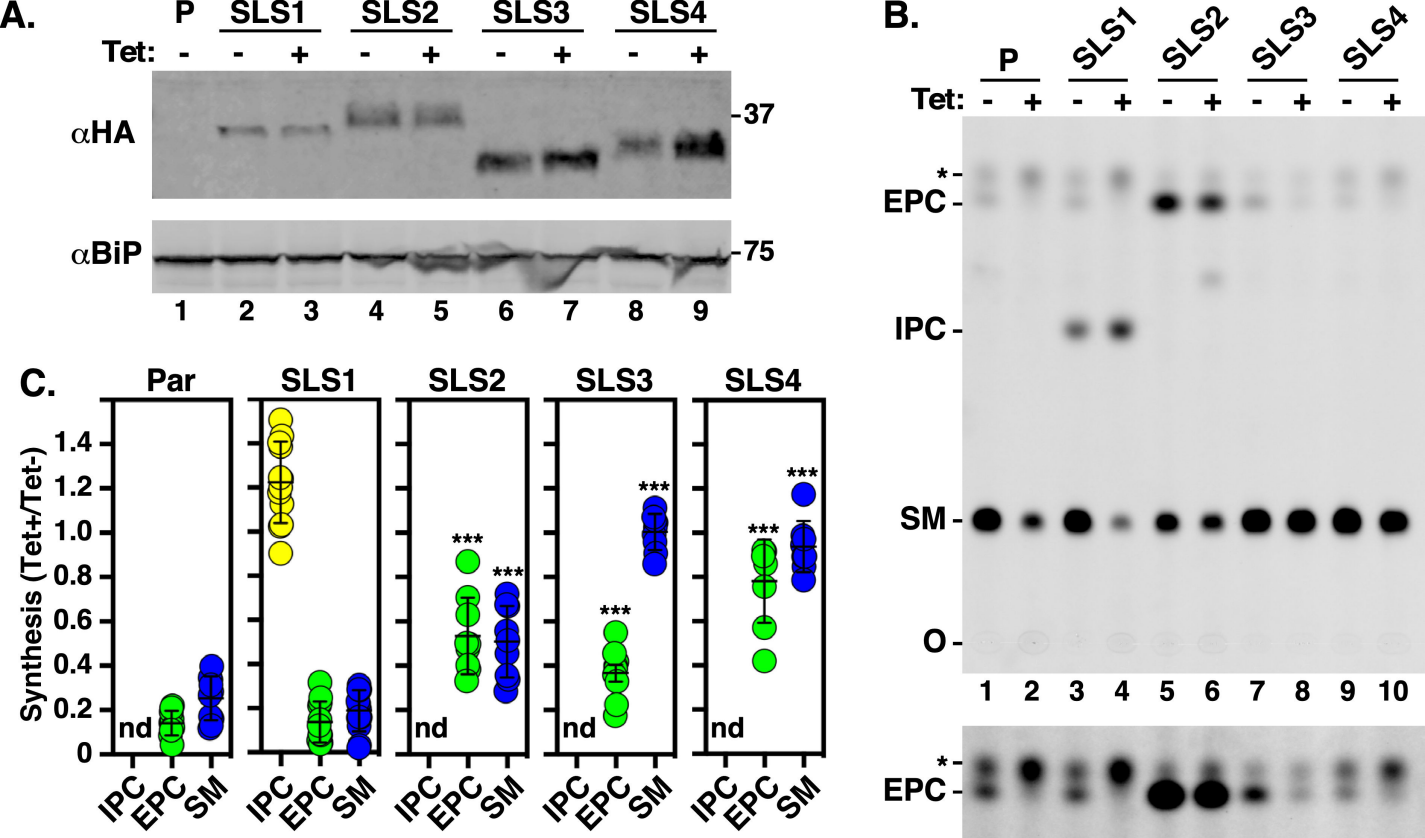
Expression and activity of RNAi^R^ *TbSLS* genes in BSF cells. The BSF SLS RNAi-resistant cell lines, as indicated, were cultured 24 h with or without silencing (Tet +/−). The parental RNAi host cell line (**P**) was used as a negative control. (**A)** Cell extracts were immunoblotted with anti-HA antibody to detect RNAi^R^ TbSLS or anti-BiP antibody as a loading control. (**B)** (Top) Cells were metabolically labeled with NBD-ceramide and lipid extracts were fractionated by normal phase TLC. The mobilities of sphingomyelin (SM), inositolphosphorylceramide (IPC), and ethanolaminephosphorylceramide (EPC) are indicated. O, origin; (*) irrelevant NBD-ceramide contaminant. (Bottom) The region containing EPC is over-enhanced. (**C)** Synthesis of SLs in each cell line was quantified and is expressed as the ratio of silenced (Tet+, RNAi^R^ only) to control (Tet−, RNAi^S^+RNAi^R^) NBD-ceramide incorporation. ***, *P* < 0.001 by one-way ANOVA relative to the equivalent parental sample. All data are expressed as mean ± std. dev. Not detected, nd.

To develop a system to investigate individual *TbSLS* gene function, we first created a parental pan-specific BSF RNAi cell line (Fig. 2A, top). Induction of dsRNA resulted in impaired *in vitro* growth at 24 h, and complete cessation at 48 h (Fig. 2C), in agreement with our previous RNAi studies (14). Paralog-specific qRT-PCR revealed individual knockdowns of 40%–50% (Fig. 2D), again in line with our previous work (~45% pan-specific knockdown). Next individual HA-tagged *TbSLS* genes, recoded to be RNAi-resistant (RNAi^R^, Fig. S2A through D) were engineered to replace a single allele of the entire *TbSLS* locus within the parental RNAi cell line (Fig. 2A, bottom). The replacement genes have 3′ UTRs derived from the aldolase intergenic region, insuring constitutive expression at housekeeping levels. Subsequent induction of RNAi silencing suppresses expression from the remaining intact allele, making cells dependent on the recombinant RNAi^R^ *TbSLS* gene for SL synthesis. In each case, induction of silencing reduced the pan-specific levels of RNAi-sensitive native transcripts (RNAi^S^) to 30%–60% of normal, while the individual RNAi^R^ transcripts were unaffected (Fig. 3A through D, right panels). It is important to note, because of the qRT-PCR strategy (see Fig. S1), that quantification of RNAi^S^ transcripts is for all four native *TbSLS* paralogs in sum, while that of RNAi^R^ transcripts is for the single recoded paralog introduced into each cell line. As expected, because SM is the major SL species in BSF trypanosomes (14) (Fig. 4, lane 1), both RNAi^R^ *TbSLS3* and *TbSLS4* genes were able to fully complement cell growth under silencing (Fig. 3C and D). Expression of RNAi^R^ TbSLS2, which makes EPC only, did not rescue growth and, in fact, enhanced lethality of silencing with complete death by 48 h (Fig. 3B). Finally, expression of RNAi^R^ *TbSLS1* failed to rescue growth, having an identical growth profile to the parental RNAi cell line (compare Fig. 3A vs Fig. 2C).

To confirm that the introduced *TbSLS^R^* genes were, indeed, constitutively expressed and functional for SL synthesis, control and silenced samples of each cell line were used for immunoblotting to detect the HA-tagged SLS proteins, and for *in vivo* SL biosynthesis assays using incorporation of fluorescent NBD-ceramide. In each case, recombinant protein of the expected sizes was detected, and these signals were unaffected by silencing of the native *TbSLS* locus, indicating that the RNAi^R^ proteins are synthesized (Fig. 4A, lanes 2–9). No tagged proteins were detected in the parental RNAi cell line (Fig. 4A, lane 1). Parental cells synthesized NBD-SM, and to a much lesser extent NBD-EPC (Fig. 4B, lanes 1 & 2), as we have observed previously (14), but this residual SM was unable to sustain cell viability (Fig. 2C). Also consistent with prior results, no IPC synthesis was detected in BSF cells. In cells expressing *TbSLS1^R^,* the same pattern of SM and EPC synthesis was observed (Fig. 4B, lanes 3 & 4), but in addition, a new RNAi^R^ species corresponding to NBD-IPC was apparent. In *TbSLS2^R^* expressing cells, greatly elevated levels of RNAi^R^ NBD-EPC synthesis were seen, as expected for this paralog (Fig. 4B, lanes 5 & 6). Finally, in cells expressing either TbSLS3 or TbSLS4, robust levels of RNAi^R^ NBD-SM synthesis, and to a lesser extent NBD-EPC, were detected (Fig. 4B, lanes 7–10). Quantifications of these data to assess the relative levels of RNAi^R^ SL synthesis are presented in Fig. 4C. Two trends should be noted. First, IPC synthesis is actually elevated in the TbSLS1^R^ cells under pan-specific silencing, as if in compensation for reduced synthesis of SM (Fig. 4C). Nevertheless, elevated IPC synthesis is unable to rescue growth of these cells (Fig. 3A). Second, while not readily apparent in the extracts of labeled TbSLS3^R^ and TbSLS4^R^ cells shown in Fig. 4B (top), significant levels of RNAi^R^ NBD-EPC are clearly seen in over-exposures (Fig. 4B, bottom), and this was consistently seen in all labeling experiments (Fig. 4C, compare Parental vs SLS3 or SLS4). Collectively, these results indicate that in each case RNAi^R^ TbSLS proteins of the expected enzymatic activity are expressed.

### TbSLS localization

**Fig 5:**
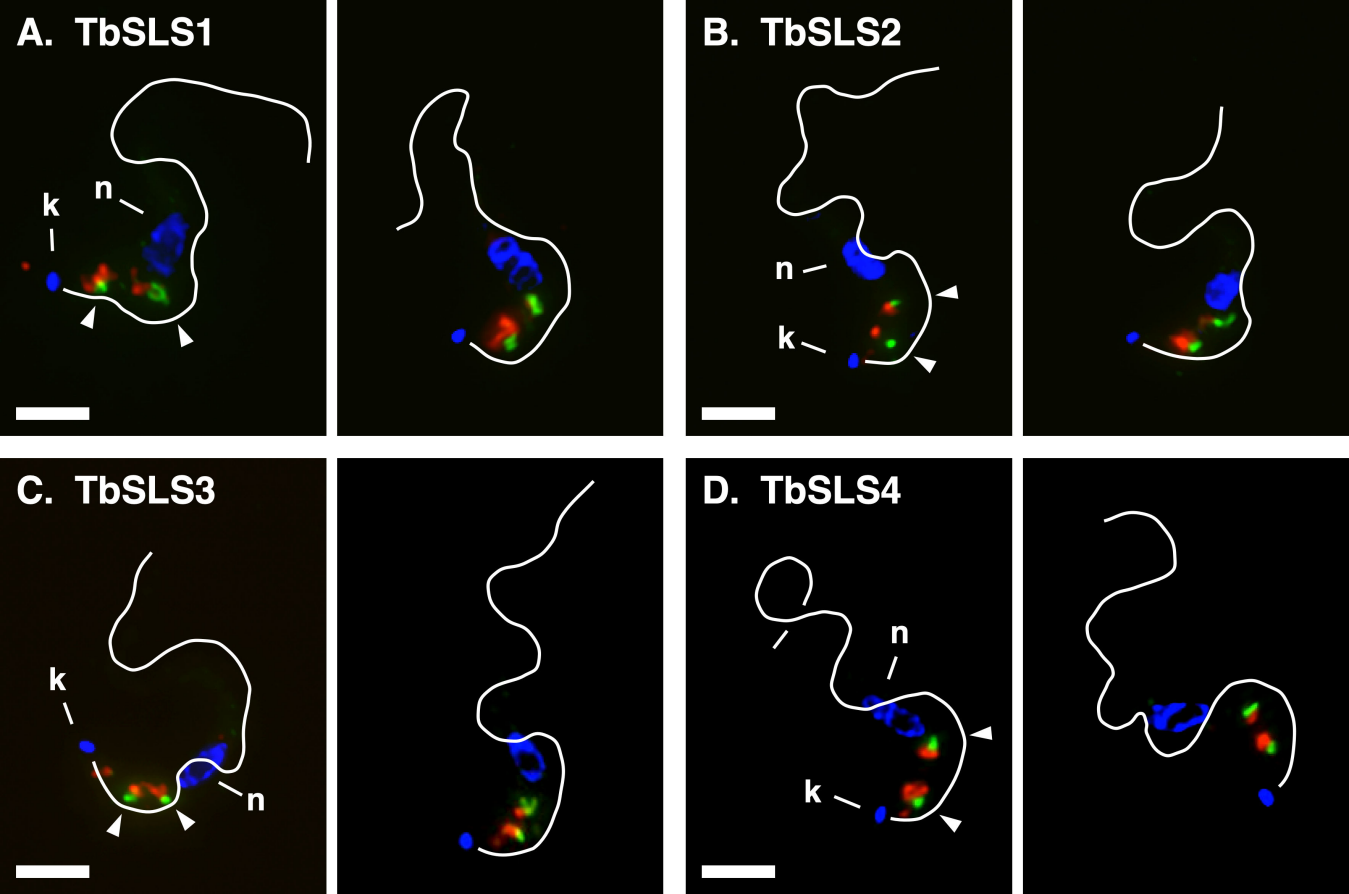
Localization of RNAi^R^ TbSLS proteins in BSF cells. The HA-tagged RNAi^R^ *TbSLS* genes were introduced into a BSF parental cell line expressing an *in situ* Ty-tagged glycosyltransferase (TbGT15:Ty) as a Golgi marker. Fixed and permeabilized cells were stained with rabbit anti-HA (red) or mAb anti-Ty (green). Nuclei (**N**) and kinetoplasts (**K**) were stained with DAPI (blue). Flagella are indicated by white lines drawn from matched DIC images. Arrowheads indicate flagellar-distal staining of TbSLSs (red) relative to GT15 (green) (left images only). Bar, 3 µm. Two independent images are presented for each cell line.

We have previously localized TbSLS4 to the Golgi in BSF trypanosomes (14), but the localization of the other paralogs is unknown. In mammalian cells, there are two SM synthase paralogs that localize to the Golgi and the plasma membrane, respectively, the former being the main source of newly synthesized SM (22, 25). Likewise, IPC synthase is found in the Golgi of yeast (26). To investigate the location of the individual HA-tagged TbSLSs, we used the same allelic replacement strategy shown in Fig. 2A in a BSF cell line that has a Ty epitope-tagged glycosyltransferase (TbGT15:Ty) as a Golgi marker (14, 27). In interphase BSF trypanosomes, there are typically two Golgi that are closely aligned with matched ER exit sites (ERES), which, in turn, are part of the flagellar attachment zone-associated ER (FAZ:ER) (Fig. S4) (28). Thus, there is a progression from external to internal of closely associated early secretory organelles. This pattern is reproduced in all the images presented in Fig. 5, as indicated by TbGT15:Ty staining (green) near the flagellum in the post-nuclear region of the cell. And as seen before, TbSLS4:HA (red) localizes in close proximity to TbGT15:Ty (green), and in an internal position from TbGT15:Ty relative to the flagellum (Fig. 5D). We interpret this to be adjacent components of the same Golgi stacks. An essentially identical pattern of staining is seen in both TbSLS2 and TbSLS3 cells (Fig. 5B and C). Finally, TbSLS1 also localizes to distal Golgi compartments when expressed in this stage (Fig. 5A). It must be noted that in some cells additional staining was seen with each tagged reporter in other areas of the post-nuclear region (not shown). This signal was variable, and in all cases, the typical Golgi-associated pattern shown in these images was also present. Overall, these results indicate that all TbSLS paralogs, including TbSLS1, which is not normally expressed at appreciable levels in BSF parasite, localize to distal Golgi compartments.

### Conditional TbSLS expression in PCF trypanosomes

**Fig 6:**
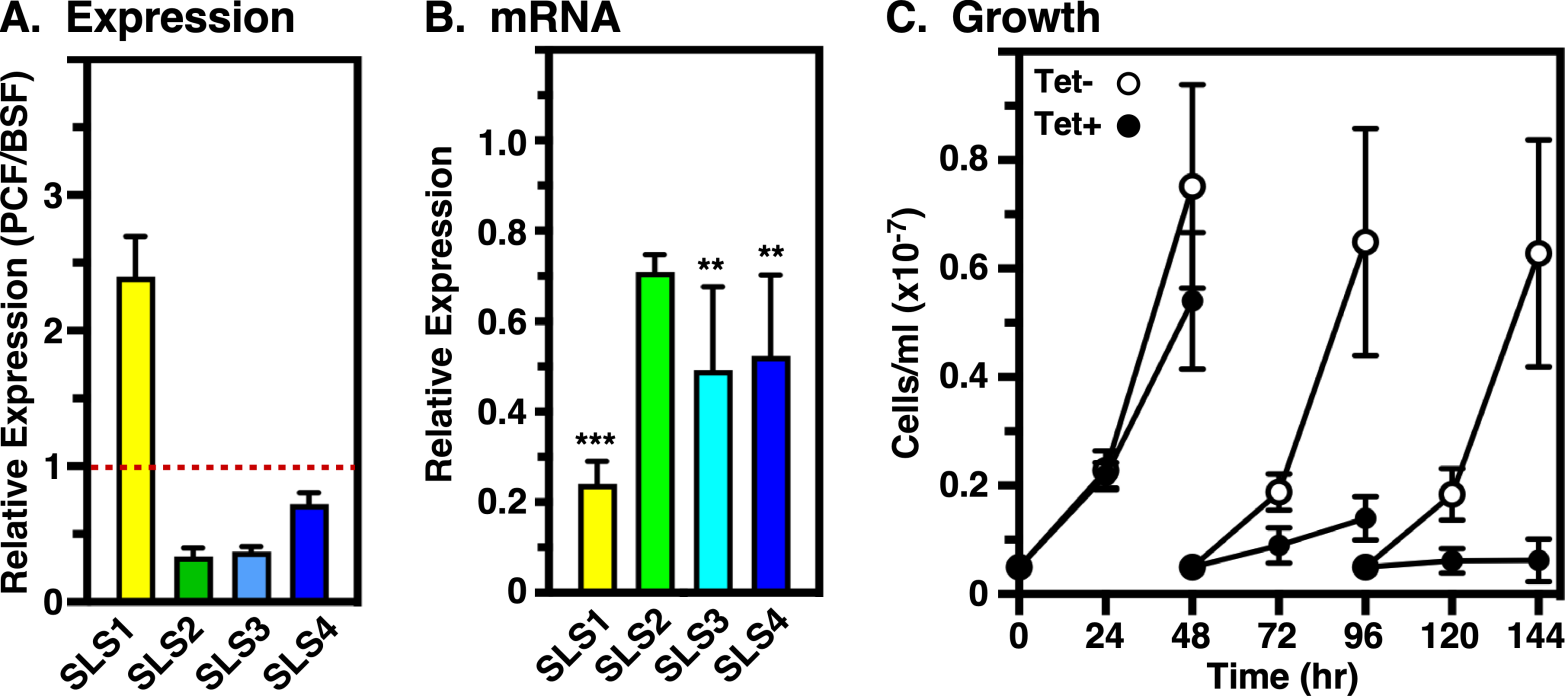
Silencing of native TbSLS in PCF trypanosomes. (A) The relative stage-specific expression levels of each paralog in parental cell lines were determined by qRT-PCR and are expressed as the ratio of BSF to PCF. Red line indicates equal expression. The relative expression between paralogs within either stage cannot be inferred from these data. These data are the inverse of those shown in Fig. 2B. (**B)** Specific mRNA levels of the individual wild-type TbSLS paralogs were determined at 24 h of silencing by qRT-PCR with primer pairs II-III (see Fig. S1; Table S1). Data are presented as the ratio of silenced (Tet+) to control (Tet−). Significance relative to control unsilenced cells calculated by one-way Anova: **, *P <* 0.005; ***, *P* < 0.001. (**C)** The parental PCF RNAi cell line with intact *TbSLS* alleles was cultured with and without tetracycline as indicated to induce pan-specific silencing. Cultures were enumerated by manual counting and adjusted to starting density (5 × 10^5^/mL) every 48 h. (**A through C)** All data are expressed as mean ± std. dev. Significance relative to control unsilenced cells calculated by one-way Anova: **, *P* < 0.005; ***, *P* < 0.001.

**Fig 7:**
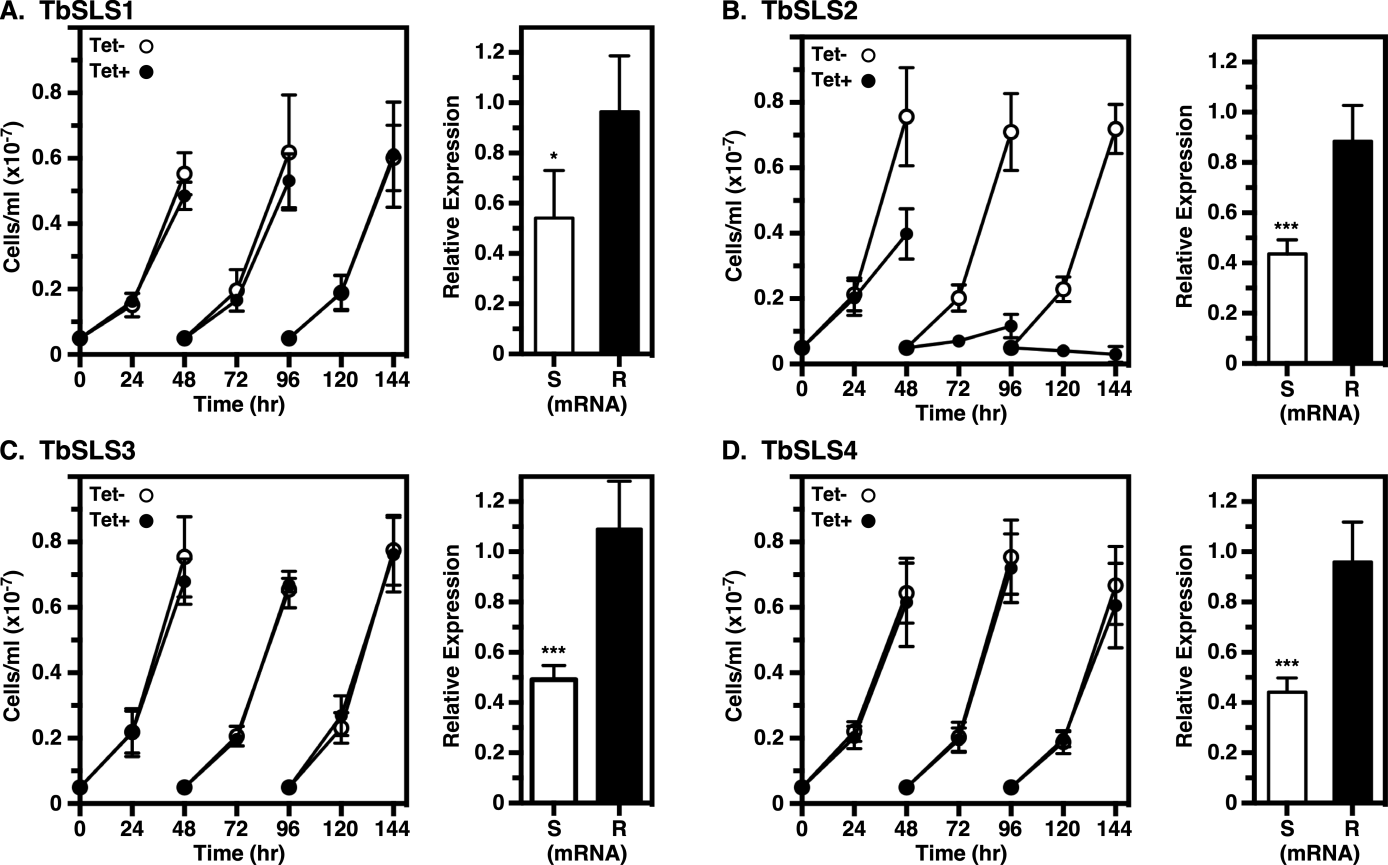
Rescue of PCF RNAi cells by RNAi^R^ TbSLS genes. PCF *TbSLS* RNAi cell lines containing RNAi-resistant *TbSLS1* (**A**), *TbSLS2* (**B**), *TbSLS3* (**C**), or *TbSLS4* (**D**) were cultured with and without tetracycline as indicated to induce pan-specific silencing of the native SLS alleles. Cell growth (left panels) was monitored as in Fig. 6C and expression levels of the native (**S**) and RNAi-resistant (**R**) genes were monitored by qRT-PCR (right panels) at 24 h with primer pairs I^S^-II and I^R^-II, respectively (Fig. S1). (**A through D)** All data are expressed as mean ± std.dev. Significance relative to control unsilenced cells calculated by one-way Anova: *, *P* < 0.05; **, *P* < 0.005; ***, *P* < 0.001.

**Fig 8:**
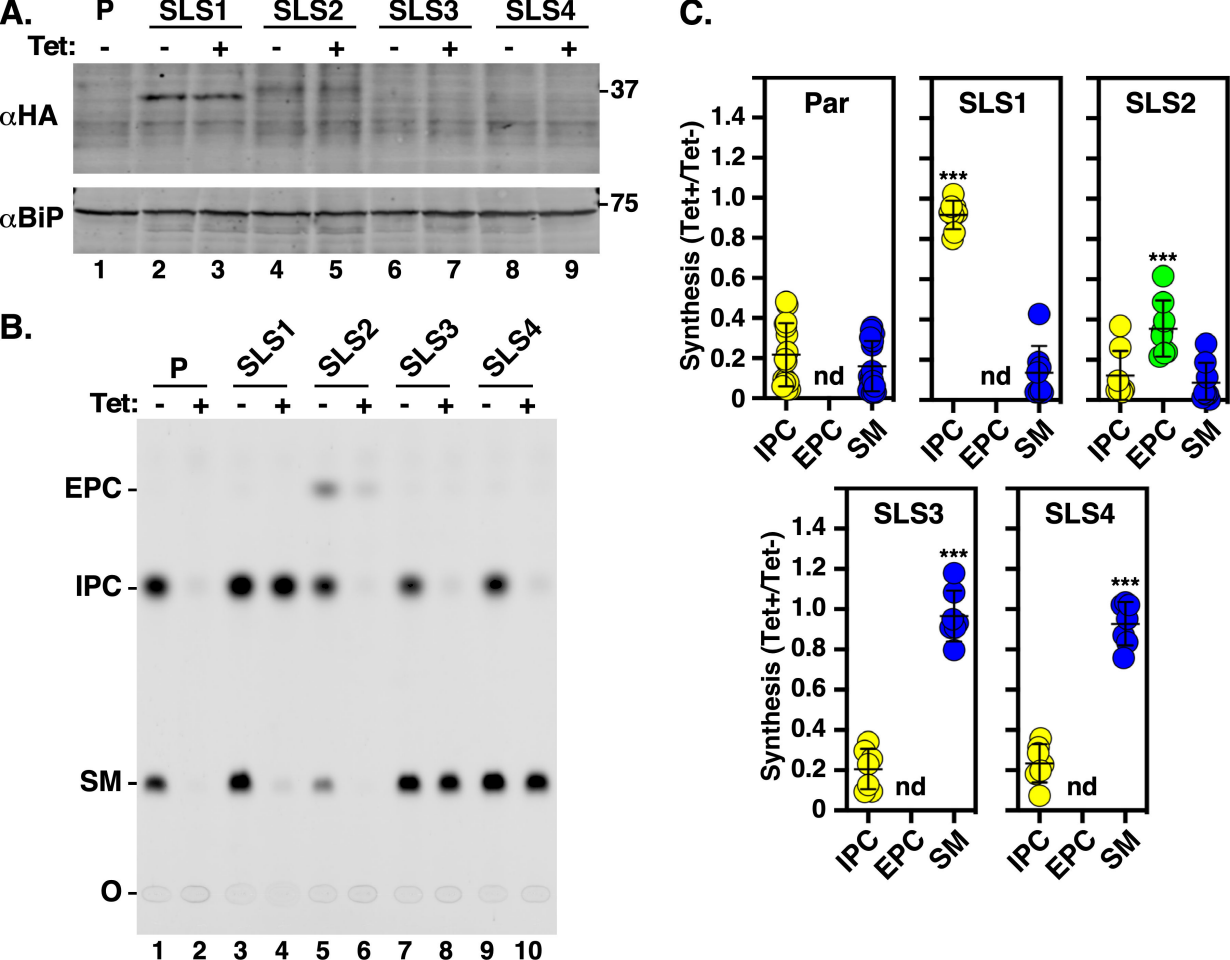
Expression and activity of RNAi^R^ TbSLS genes in PCF cells. The PCF SLS RNAi-resistant cells lines, as indicated, were cultured 24 h with or without silencing (Tet −/+). The parental RNAi host cell line (**P**) was used as a negative control. (**A)** Cell extracts were immunoblotted with anti-HA antibody to detect RNAi^R^ TbSLS or anti-BiP antibody as a loading control. (**B)** Cells were metabolically labeled with NBD-ceramide and lipid extracts were fractionated by normal phase TLC. The mobilities of sphingomyelin (SM), inositolphosphorylceramide (IPC), and ethanolaminephosphorylceramide (EPC) are indicated. O, origin. (**C)** Synthesis of SLs in each cell line was quantified and is expressed as the ratio of silenced (Tet+, RNAi^R^) to control (Tet−, RNAi^S^+RNAi^R^) NBD-ceramide incorporation. ***, *P* < 0.001 by one-way ANOVA relative to the equivalent parental sample. All data are expressed as mean ± std. dev. Not detected, nd.

We employed the same strategy used above to investigate TbSLS function in PCF parasites. In wild-type cells, *TbSLS1* expression is ~2.5× higher in PCF than BSF cells, while the expression of *TbSLS2-4* is considerably lower (Fig. 6A). In the parental RNAi cell line, induction of pan-specific dsRNA synthesis (24 h) resulted in an ~75% reduction of *TbSLS1* mRNA, and ~50% reductions in *TbSLS2-4* (Fig. 6B). Consequently, after 24 h of silencing cell growth slowed markedly and ceased entirely by day 4 (Fig. 6C). Again, we assessed the ability of individual RNAi^R^ *TbSLS* genes to rescue growth under silencing of the endogenous allele. Similar to BSF cells, pan-expression of the combined native paralogs was reduced 50%–60%, while the individual RNAi^R^ transcripts were unaffected (Fig. 7A through D, right panels). As expected, *TbSLS1* expression restored growth to normal levels (Fig. 7A, left). However, *TbSLS2* was unable to rescue growth, albeit without the enhanced lethality seen in BSF parasites with sole expression of this paralog (Fig. 7B, left). Immunoblot analyses of both cell lines indicated RNAi^R^ synthesis of proteins of the expected sizes for both TbSLS1 and TbSLS2 (Fig. 8A, lanes 2–5). In each case, NBD-ceramide labeling revealed RNAi^R^ production of the appropriate SL, NBD-IPC for TbSLS1 and NBD-EPC for TbSLS2 (Fig. 8B, lanes 3–6, & Fig. 8C). Somewhat unexpectedly, both *TbSLS3* and *TbSLS4* expression fully rescued growth of PCF cells (Fig. 7C and D, left); however, no signal was seen for either TbSLS3 or TbSLS4 protein (Fig. 8A, lanes 6–9), presumably due to low expression levels. Nevertheless, both cell lines displayed robust RNAi^R^ synthesis of NBD-SM (Fig. 8B, lanes 7–10, Fig. 8C) confirming the presence of active proteins and consistent with the growth rescue phenotype. The quantitative NBD:SL synthesis data from BSF (Fig. 4C) and PCF cells (Fig. 8C) are presented together in Fig. S3 for direct comparison.

**Fig 9:**
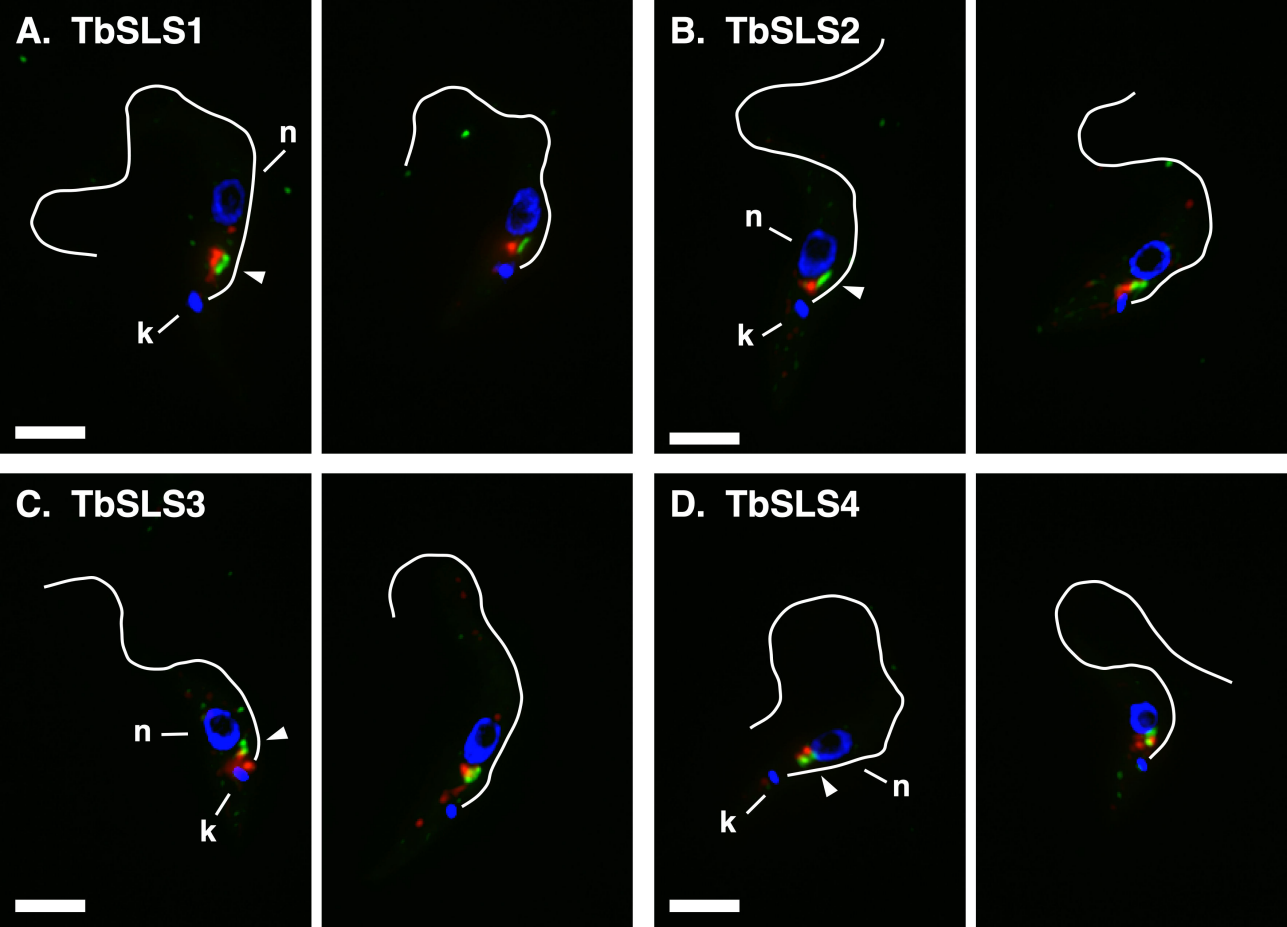
Localization of RNAi^R^ TbSLS proteins in PCF cells. As indicated, the PCF HA-tagged RNAi^R^ TbSLS were fixed and permeabilized cells and stained with mouse anti-HA (red, TbSLS) or rabbit anti-TbGRASP (green, Golgi). Nuclei (**N**) and kinetoplasts (**K**) were stained with DAPI (blue, left images only). Flagella are indicated by white lines drawn from matched DIC images. Arrowheads indicate flagellar-distal staining of TbSLSs (red) relative to TbGRASP (green) (left images only). Bar, 3 µm. Two independent images are presented for each cell line.

Finally, we determined the localization of each TbSLS paralog in PCF cells. In contrast to BSF cells, interphase PCF trypanosomes have just one post-nuclear ERES:Golgi junctional complex (27, 29, 30), albeit with the same basic arrangement (Fig. S4). For these studies, we used the PCF SLS RNAi cell line bearing the individual tagged *TbSLS* genes and used anti-TbGRASP antibodies as a Golgi marker (27, 29). In each case, TbGRASP staining (green) detected a single Golgi in close proximity to the FAZ, each of which had a closely associated TbSLS signal (red), consistent with localization in distal Golgi compartments (Fig. 9A through D). Again, there was variable staining in other post-nuclear regions (not shown), but always the Golgi localization was consistent.

## DISCUSSION

We have performed detailed functional analyses of the four SL synthases in *T. brucei*. Alone of all kinetoplastids, indeed of all eukaryotes, *T. brucei* is the only species proven to synthesize all three of the major PSL species—IPC, SM, and EPC. All other kinetoplastid protozoa, excluding the African salivarian lineage (discussed below), have a single syntenic SLS gene (24). Based on the presence of the “signature” serine residue, all of these are either proven or predicted IPC synthases. Mammalian cells are the only other system studied thus far in which multiple SLS genes have been characterized (22, 25, 31–34). Three SLSs have distinct functions based on enzymatic specificity and intracellular localizations. SMS1 is responsible for *de novo* synthesis of SM/EPC in the Golgi, SMS2 regenerates SM from ceramide derived from turnover in the plasma membrane, and SMSr synthesizes EPC in the ER. In contrast, all four TbSLSs localize to distal sub-compartments of the Golgi in both BSF and PCF trypanosomes. This eliminates the possibility that any differences in function can be ascribed to distinct SLS localizations.

The role of the PSLs in trypanosomes differs markedly from the other human infectious kinetoplastid parasites, *T. cruzi* and *L. major*, both of which contain single orthologous genes for IPC synthase. Disruption of the *T. cruzi* gene had minor effects on *in vitro* growth of insect stage epimastigotes, but differentiation to infectious metacyclic forms, *in vitro* intracellular growth as amastigotes, and subsequent differentiation to culture-derived trypomastigotes were all significantly impaired (35). Knockouts were unable to establish meaningful infection *in vivo*. In *L. major*, knockout eliminated detectable IPC but had only a minor effect on *in vitro* promastigote cell growth and differentiation to infectious metacyclics (36). Intracellular amastigotes were likewise viable, contained significant amounts of SM scavenged from host cells, and surprisingly had enhanced virulence *in vivo*. The later result suggests that the presence of IPC dampens or limits *in vivo* growth, which would be salubrious for maintaining prolonged infection. It must be noted, however, that IPC synthase knockout in *L. mexicana* likewise had no effect on *in vitro* growth, but was markedly detrimental to *in vivo* pathogenesis, suggesting species-specific differences in IPC utilization (37). Thus, *T. cruzi* does not need IPC for insect stage proliferation (although actual infection of reduviid vectors was not tested), but needs IPC for both *in vitro* and *in vivo* growth and development of mammalian stages, while *L. major* can dispense with IPC throughout its life cycle, although again, infection of the sand fly vector was not attempted. In contrast, TbSLSs are essential for viability in both BSF and PCF trypanosomes, confirming our previous results in BSF cells (14), extending them to the insect stage, and presumably to the entire life cycle, which includes additional stages in the tsetse fly vector. Although orthologous, the wide evolutionary gulf between mammalian and kinetoplastid SLSs has fueled legitimate discussion of the potential for developing chemotherapeutic agents targeting the latter (8). The complete lack of the need for IPC in *L. major* would seemingly rule out IPC synthase as a drug target in this group of parasites. However, the dependence on IPC for *T. cruzi* and *L. mexicana* pathogenesis in the mammalian host, and the utter essentiality of PSL synthesis in *T. brucei*, do hold out the potential for future drug development with these parasites.

This situation in *T. brucei* has allowed us to develop an RNAi complementation system to test in isolation the ability of each TbSLS to rescue lethality when expressed constitutively in RNAi^R^ form. These analyses were performed in parallel in both the BSF and PCF stages of the life cycle. In BSF trypanosomes, as expected, both TbSLS3 and TbSLS4 fully complemented the RNAi phenotype. *TbSLS2* expression was not detrimental in control cells but failed to rescue RNAi lethality. In fact, sole dependence on EPC synthesis markedly exacerbated the lethal RNAi phenotype in BSF cells. Finally, constitutive expression of *TbSLS1*, with concomitant IPC synthesis, was not detrimental to BSF cells in culture; however, it too failed to rescue RNAi lethality in the absence of SM synthesis. Collectively, these results indicate that BSF cells are critically dependent on SM to fulfill the requirement for PSLs—it cannot be replaced by IPC or EPC. Conversely, in PCF cells, TbSLS1 alone fully complemented the RNAi phenotype indicating that IPC is sufficient for all PSL-dependent functions. TbSLS2 did not rescue viability although without the enhanced lethality seen in BSF cells. Most surprisingly, both TbSLS3 and TbSLS4 fully rescued PCF cells from RNAi-mediated lethality. Thus, while IPC is the major PSL species in PCF cells, it is not essential for viability *in vitro*. Apparently, EPC/SM alone is sufficient to carry out all PSL-mediated functions, raising the question of why IPC synthesis is maintained in PCF cells (discussed below). Finally, it is also worth noting that these studies fully confirm our prior assignment of enzymatic specificity for each TbSLS.

The implications for the diversification of the *SLS* genes in the larger group of salivarian trypanosomes are striking. *T. vivax*, near the base of this lineage (Fig. 1), has one *SLS* gene encoding a bifunctional SM/EPC synthase. Subsequently diverging clades have amplified the SLS locus to contain multiple SLS genes of different proven or inferred enzymatic specificity. Both *T. brucei* (four genes, proven) and *T. congolense* (three genes, inferred) have maintained IPC synthesis, which is developmentally regulated in *T. brucei*. In contrast, *T. suis* (three genes, inferred), the least studied salivarian and closest to *T. brucei*, has not retained an apparent IPC synthase activity. Thus, *T. vivax* and *T. suis* are the only kinetoplastids to not require IPC. We have proposed a reasonable pathway for this diversification (24). The first step is a mutation in an IPC synthase-containing ancestor (Fig. 1, red dot) leading to *T. vivax* with a single SM/EPC synthase. This would be followed by independent amplification and diversification in the branches leading to the other clades such that IPC and SM/EPC synthases are both present. Lastly is the loss of IPC synthase in the *T. suis* branch. An alternative scenario, which seems less likely, invokes the conversion of a single IPC synthase gene to SM/EPC synthase in a basal salivarian ancestor (Fig. 1, red dot) followed by amplification in the later diverging clades. This pathway would require subsequent independent reversion of one *SLS* gene to IPC synthase activity in both *T. brucei* and *T. congolense*.

It is of course not possible to know the selection pressures that led to this unique diversification in the *Salivaria*. Nevertheless, based on the situation with the proven SLSs in *T. brucei* and *T. vivax,* we have proposed a simple model (24), central to which is that the salivarians, alone among the human infectious kinetoplastids, do not replicate intracellularly in the mammalian host—they replicate freely in the blood and tissue spaces. This suggests that IPC may be detrimental, perhaps by “alerting” host innate immunity. Relevant to this may be the aforementioned observation that loss of IPC synthesis enhances pathogenesis in *Leishmania major* (36), albeit as an intracellular pathogen. Second, *T. vivax* has very limited development in the tsetse vector, never progressing beyond the proboscis and lacking the more complex stages in the midgut, proventriculus and salivary glands found in *T. brucei* (38). Perhaps more complex development in the tsetse requires IPC, as with all other kinetoplastid parasites, and hence, *T. vivax* is able to eliminate IPC synthase. There are admittedly several problems with this model. First, *T. suis*, which is closely related to *T. brucei* and has its own complex life cycle in the tsetse (39), seemingly has also dispensed with IPC. Second, *T. theileri*, a distantly related stercorarian (*T. cruzi* related) bovine parasite (Fig. 1), only has IPC synthase (predicted) yet replicates extracellularly in the mammalian host (40, 41). These issues aside, the model does frame relevant experimentally amenable questions in *T. brucei*. We have shown that IPC synthesis is not detrimental to the growth of BSF trypanosomes *in vitro*, but will this hold for *in vivo* infection? Conversely, we have shown that IPC synthesis is not required by PCF cells in culture, but will this be the same in the tsetse fly? Answers to these questions will challenge the model, but whatever the outcome will also further our understanding of the unusually complex biology of PSLs in trypanosomes.

## MATERIALS AND METHODS

### Parasite culture and construction of cell lines

The Lister 427 strain of *Trypanosoma brucei brucei* was used in all *in vitro* experiments. The tetracycline-responsive single marker BSF 13–90 and PCF 29–13 cell lines were used for RNAi (42). These cell lines constitutively express T7 polymerase and Tet repressor proteins. BSF cells were maintained in HMI9 media supplemented with 5 µg/mL G-418 and 10% Tet-free FBS (Clontech, Mountain View, CA) at 37°C and 5% humidified CO_2_ (43). For localization studies in BSF cells, an *in situ* Ty epitope tag (EVHTNQDPLD) was fused to the C-terminus of the endogenous β-*N*-acetylglucosaminyl transferase II gene *TbGT15* (Tb927.7.300) (44) as described in reference (14). Its Golgi localization was confirmed previously in BSF (14, 28). Clonal cell lines were obtained by limiting dilution in the presence of 5 µg/mL G-418 and 0.4 µg/mL puromycin, and the best GT15:Ty expresser was selected by immunoblot and IFA (not shown). PCF cells were maintained in Cunningham’s medium with 50 µg/mL hygromycin, 25 µg/mL G-418, and 10% Tet-free FBS at 27°C (45).

A pan-specific *TbSLS1-4* RNAi construct was generated in the pLEW100v5X:Pex11 stem loop vector (46). Using Lister 427 strain genomic DNA, an 831 bp fragment (nt 1–831) of the *TbSLS1* ORF (Tb927.9.9410) was PCR amplified with nested 5′ HindIII/XbaI and 3′ XhoI/NdeI sites using primers SLS-RNAiF and SLS-RNAiR (Table S1). The amplicon was sequentially inserted upstream of the Pex11 stuffer of pLEW100v5X:Pex11 stem loop vector using HindIII/XbaI, and downstream in the opposite orientation using XbaI/NdeI. The sequence was confirmed using primers pLEW100F and ALDOINTR (Table S1), and the resultant plasmid was linearized with NotI for transfection of the BSF 13–90 GT15:Ty and the PCF 29–13 cell lines. Transfection was performed by electroporation as described previously (28, 47). Clonal cell lines were obtained by limiting dilution and selection with phleomycin (4 µg/mL). Induction of pan-specific *TbSLS* dsRNA was achieved by the addition of 1 µg/mL of tetracycline.

RNAi-resistant (RNAi^R^) versions of each *TbSLS1-4* orf with recoded RNAi target regions and N-terminal in frame HA tags (YPYDVPDYA) were synthesized (Integrated DNA Technologies, Coralville, IO) in order to replace one allele of the *TbSLS1-4* locus by homologous recombination as shown in Fig. 2A. Recoding altered codons (nt 1–831, see Fig. S2 for wild type vs recoded sequence alignments) to the next most frequently used codon in *T. brucei* housekeeping genes (48, 49), and the remaining 3′ orfs were wild type. The allelic replacement constructs assembled in our pXS6 vector (46) contained (5′−3′): 5′ UTR targeting region (nts −634 to −1; relative to the *TbSLS1* start codon); *HA:TbSLS^R^*; aldolase intergenic region; hygromycin resistance gene; 3′ UTR targeting region (nts 1–707; relative to the *TbSLS4* stop codon). The 5′ and 3′ targeting sequences were amplified from Lister 427 strain DNA using primer pairs 5′UTR-SLS1F/5′UTR-SLS1R primers 3′UTR-SLS4F/3′UTR-SLS4R (Table S1), respectively. Constructs were confirmed by sequencing using primers listed in Table S1. The constructs were linearized with ClaI/XbaI for homologous replacement of one allele of the endogenous *TbSLS1-4* locus of the pan-specific TbSLS1-4 RNAi cell lines (Fig. 2A). For replacements in PCF cells, the hygromycin-resistant cassette was exchanged for puromycin-resistance. Transfections and clonal selections were as described above, but using the regular antibiotics plus hygromycin (5 µg/mL) for BSF and puromycin (10 µg/mL) for PCF.

### Quantitative reverse transcription polymerase chain reaction

Specific transcript levels were determined using quantitative RT-PCR (qRT-PCR). Total RNA was isolated from log phase cultures using RNeasy Mini kit (Qiagen, Valencia, CA). RNA was treated with DNAse1 on-column using RNase-Free DNase Set (Qiagen), and cDNA was synthesized using iScript cDNA synthesis kit (BioRad, Hercules, CA). qRT-PCR was performed using diluted cDNAs and Power SYBR green PCR Master Mix (Life Technologies, Carlsbad, CA) with oligonucleotide pairs specifically targeting transcripts from each of the wild-type *TbSLS* genes (Fig. S1, primer pairs II-III), or pan-specifically for all wild type or RNAiR genes (Fig. S1, primer pairs I-II). All primer sequences are in Table S1. TbZFP3 (Tb927.3.720, nts 241–301) was used as the control amplicon (50, 51). qRT-PCR efficiency was calculated as 10^-1/slope^-1 with the logarithm of the template concentration on the *x* axis and the C_T_ plotted on the *y* axis. A qPCR efficiency of 100% thereby indicates that the amount of PCR product doubles with each cycle (52). Amplification was performed using an Applied Biosystems StepOne Real-Time PCR System (Life Technologies, Carlsbad, CA). For each transcript, post-amplification melting curves indicated a single dominant product. Transcript levels were normalized to TbZFP3 as endogenous control. All calculations and normalizations were done using StepOne software, version 2.2.2. Reactions were performed in triplicate or sextuplicate, and means ± standard deviations for at least three biological replicates are presented.

### Immunoblotting

Gels were transferred to Immobilon-P membranes (Millipore Corp., Bedford, MA) using a Trans-Blot Turbo apparatus (BioRad, Hercules, California). Membranes were blocked with PBS, 150 mM NaCl, 0.5% Tween20 (PBS-T) containing 10% defat milk, washed and probed with appropriate dilutions of primary, and with secondary antibodies in Odyssey Blocking Buffer (Li-Cor Biosciences, Lincoln NE) diluted 1:10 in PBS-T. Primary antibodies were monoclonal mouse anti-HA used at 1:1,000 (Clone HA-7, Sigma-Aldrich, St. Louis, MO) and polyclonal rabbit anti-BiP used at 1: 5,000 (53). Secondary antibodies were goat anti-mouse IgG:IRDye680RD and goat anti-rabbit IgG:IRDye800CW used at 1: 10,000 (Li-Cor). All washes were with PBS-T. Quantitative fluorescent signals were scanned on an Odyssey CLx Imager (Li-Cor).

### *In vivo* metabolic labeling with fluorescent ceramide

Trypanosomes from log phase cultures were collected, washed, and suspended at 2–5 × 10^7^/mL in serum-free Cunningham’s (PCF) or HMI9 (BSF) media containing 5 µM NBD C6-ceramide complexed to BSA (Molecular Probes, Seattle, WA) and were incubated on ice for 30 min. Cells were then washed to remove free BSA:ceramide complex and incubated in serum-free media at 27°C (PCF) or 37°C (BSF) for 120 min to allow metabolism of the fluorescent ceramide. Total lipids were extracted with CMW (10:10:3, by v), dried under N_2_, and subjected to *n*-butanol/water partitioning. Lipids recovered from the butanol phase were fractionated by thin-layer chromatography (TLC) in chloroform:methanol:acidic acid:water (25:15:4:2, by v) on glass TLC Silica Gel 60 plates (Merck, Gibbsburg, NJ). Quantitative fluorescent lipid bands were captured on a ChemiDoc Imaging System using Image Lab Software (Biorad, Hercules, CA).

### Data analyses

Fluorescent immunoblots and TLC scans were quantified with ImageJ software (http://imagej.nih.gov/ij/). For analysis of specific band intensities, signals were corrected by subtraction of the signal from equivalent unlabeled areas of each lane. All subsequent data management was performed with Prism5 software (GraphPad Software, Inc., San Diego CA).

### Epifluorescence microscopy

Cultured parasites were prepared for immunofluorescence microscopy as described previously (54). Primary antibodies were rat anti‐HA (1:250, Sigma-Aldrich, St. Louis, MO) to stain HA:TbSLS^R^ in BSF and PCF, monoclonal mouse anti-Ty used at 1:500 to stain the Ty:GT15 in BSF Golgi (UAB Hybridoma Facility, Birmingham, AL), and polyclonal rabbit anti-GRASP used at 1:500 to stain the Golgi in PCF (29). Serial 0.2 mm image Z‐stacks were collected from stained cells with capture times from 100/150 to 400/500 ms (100X PlanApo, oil immersion, 1.46 na) on a motorized Zeiss AxioImager M2 equipped with a rear‐mounted excitation filter wheel, a triple pass (DAPI/FITC/Texas Red) emission cube, and differential interference contrast optics. All images were captured with an Orca AG CCD camera (Hamamatsu, Bridgewater, NJ) in Volocity 6.0 acquisition software (Improvision, Lexington, MA), and individual channel stacks were deconvolved by a constrained iterative algorithm, pseudocolored, and merged using Volocity 6.0 restoration software. All images presented are summed‐stack projections of deconvolved individual or merged channels unless otherwise stated in the text.

## SUPPLEMENTAL MATERIAL

Supplemental material mbio.03501-24-s0001.pdf Supplemental table and figures.

ASM does not own the copyrights to Supplemental Material that may be linked to, or accessed through, an article. The authors have granted ASM a non-exclusive, world-wide license to publish the Supplemental Material files. Please contact the corresponding author directly for reuse.
