# Supplementary material for: Stage-specific function of sphingolipid synthases in African trypanosomes
Source: mBio. 2024 Dec 16;16(2):e03501-24. doi: 10.1128/mbio.03501-24 (PMC11796370; doi:10.1128/mbio.03501-24)
Supplement: Supplemental material — Supplemental table and figures. [file mbio.03501-24-s0001.pdf]

SUPPLEMENTAL DATA

Stage-specific Function of Sphingolipid Synthases in African Trypanosomes

Norton Heise[1], Carolina M. Koeller[1], Mohamed Sharif[2,3] and James D. Bangs[2,*]

Table S1

Primer	Sequence	GC (%)	Tm (ºC)	Database
**Mutagenesis of pJB842 to change *SacI* into *XbaI*[1]**				
pJB842SacIXbaIF	5'-GGTACAAGACTGtctagaCAATTCGCCCTATAG-3'	45.5	60.4	JB2095
pJB842SacIXbaIR	5'-CTATAGGGCGAATTGtctagaCAGTCTTGTACC-3'	45.5	60.4	JB2096
***TbSLS1-4* pan-RNAi[1]**				
SLS-RNAiF	5'-ACaagctttctagaATGGCTGTCCCACCAGTGG-3'	51.5	65.5	JB2097
SLS-RNAiR	5'-CActcgagcatatgATCAGCGTTGTGACCTACTG-3'	50.0	63.6	JB2098
**Cloning *TbSLS1-5'-UTR* into *ClaI* and *EcoRI* of pJB842XbaI[1]**				
5'UTR-SLS1F	5'-CTatcgatGTAAATGCGGCAACGATAGG-3'	46.4	59.2	JB2099
5'UTR-SLS1R	5'-CTgaattcTGGTGGTGGTACTAATCCTGG-3'	48.3	60.0	JB2100
**Cloning *TbSLS4-3'-UTR* into *PacI* and *XbaI* of pJB842XbaI[1]**				
3'UTR-SLS4F	5'-CAttaattaattGTTTCATATATATCTATATAGCTTTG-3'	18.4	51.7	JB2101
3'UTR-SLS4R	5'-CAtctagaCGCTAGTCTCTTGGTGTTTCG-3'	48.3	59.8	JB2102
**Sequencing and/or PCR**				
HYGROF	5'-GTACTCGCCGATAGTGGAAAC-3'	52.4	55.2	JB1278
ALDOINTR	5'-CACGGAAAAGACACGAGACA-3'	50.0	55.0	JB1780
SLS14FR	5'-CTCAGGCAGTTTCTGGAATCG-3'	52.4	55.8	JB2110
SLS14RR	5'-CCTCTCGTGGGTTATCTGGAGT-3'	54.5	57.9	JB2111
SLS14F	5'-CAGAATGAGGAAACCCTTGCCAT-3'	47.8	57.6	JB2112
SLS14R	5'-GATCTGGTAGCGGCTTCGTC-3'	60.0	57.8	JB2113
5UTRSLS1	5'-CCATTGGATGTAGGACGAACAC-3'	50.0	55.6	JB2125
3UTRSLS4	5'-CCAACACACAGGCCGAATA-3'	52.6	55.2	JB2126
**Real time qPCR[2]**				
SLS-SF (I, dark green)	5'-CATAGCAGTAGGTCACAACGCTG-3'	52.2	57.7	JB2343
SLS-RF (I, light green)	5'-GTTTATCGCGGTCGGCCATAAT-3'	50.0	57.7	JB2344
SLS-F (II, dark green)	5'-CCATGTTGTGGTGCCAATTC-3'	50.0	54.9	JB2120
SLS-R (II, red)	5'-GAATTGGCACCACAACATGG-3'	50.0	54.9	JB2345
SLSR1 (III, red)	5'-CTATCGTCCCTCTCTCGTAACT-3'	50.0	55.0	JB2121
SLSR2 (III, red)	5'-TTCCCTCGAAATTCATCATTTCTTC-3'	36.0	53.6	JB2122
SLSR3 (III, red)	5'-CATCTACGACTTTCCTGCTAGAC-3'	47.8	54.6	JB2123
SLSR4 (III, red)	5'-CCAGCACACCATTCATTTCATC-3'	45.5	54.6	JB2124

[1]. Restriction sites in lower case.

[2]. Primer location and color is indicated I, II, or III in accordance with the diagram in Fig. S1.

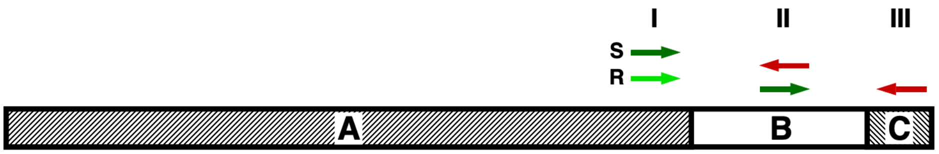

Figure S1. PCR Strategy. A diagram (not to scale) of the TbSLS orf. **A.** Common amino acid sequence of the RNAi target region that is found in the wild type (RNAi-sensitive) and recoded (RNAi-resistant) versions of each TbSLS paralogue. **B.** Common wild type amino acid sequence found in all *TbSLS* paralogues. **C.** Divergent C-terminal amino acid sequences found in each individual paralogue. **I.** Position of the specific wild type (SLS-SF, dark green, $RNAi^{S}$) and recoded (SLS-RF, light green, $RNAi^{R}$) forward primers used in Figs. 2 & 7 **II.** Position of reverse (SLS-R, red) and forward (SLS-F, green) primers used in Figs. 2 & 7, and Figs. 1 & 6, respectively. **III.** Position of unique reverse primers (SLSR1, SLSR2, SLSR3, SLS4, red) used in Figs. 1 & 6. All primers are defined in Table S1.

Figure S2A-D. Alignments of native and RNAiR TbSLS sequences. Each native wild type TbSLS sequence (top line) is aligned to its engineered RNAi-resistant counterpart (middle line) from the N-terminal start codon (highlighted green) of the native orf to the C-terminal stop codon (highlighted red). Translated sequences are shown (bottom line). All numberings are relative to the first base pair/amino acid of these sequences. The region of each orf targeted for pan-specific RNAi silencing is shaded grey. Recoded sequences in red capital letters indicate base pairs changed in the engineered RNAi-resistant sequences. Highlighted amino acid residues are the conserved SLS HDD catalytic triad (red), and the experimentally determined Ser/Phe residue (purple) that controls *T. brucei* catalytic specificity. The paralogue-specific divergent C-termini of each TbSLS are indicated by colored coded highlighting (TbSLS1, yellow; TbSLS2, green; TbSLS3, light blue; TbSLS4, blue). In frame N-terminal HA tag sequences are highlighted (fuschia). The positions of the various PCR primers used in this work are underlined and labeled I, II, or III in accordance with the diagram in Fig. S1. The forward Set I primers are specific to wild type (RNAi sensitive) or recoded (RNAi resistant) sequences of each TbSLS paralogue. Primers are defined in Table S1. EcoRI, NheI and XmaI restriction sites used for cloning in the modified plasmid pJB842, as described in Materials & Methods, are indicated (italics, underlined).

Fig S2A. TbSLS1 (IPC synthase) (Tb927.9.9410)

```
                                                  EcoRI          HA EPITOPE         NheI
                                                 gaattcATGtacccatacgacgtcccagattacgcggctagc
                                                       M  Y  P  Y  D  V  P  D  Y  A  A  S

SLS1_WT    atggctgtcccaccagtggaaatgtatagtggttccttttggaacagaatgaggaaacccttgccattacgaacg   75
SLS1_R     atggcGgtTccGccGgtTgaGatgtaCTCAggCAGTttCtggaaTCgGatgCgAaaGccACtCccGCtCAgGacA
            M  A  V  P  P  V  E  M  Y  S  G  S  F  W  N  R  M  R  K  P  L  P  L  R  T   25

SLS1_WT    caagtcattcgattcacagttgtattcgtaatcgtctctttcatccttgcggttgcgttgcaaatcacacatgaa   150
SLS1_R     caGgtAatCAgGttTacCgtGgtCttTgtCatAgtAAGCttTatATtGgcAgtGgcACtCcaGatAacCcaCgaG
            Q  V  I  R  F  T  V  V  F  V  I  V  S  F  I  L  A  V  A  L  Q  I  T  H  E   50

SLS1_WT    cgtatgccggaccccaaggtgacgaagccgctaccagatctcggttttgaacttttgacgaagatatcatttctc   225
SLS1_R     AgGatgccAgaTccAaaAgtTacAaaAccATtGccGgaCTtAggCttCgaGTtGCtCacAaaAatCAGCttCTtG
            R  M  P  D  P  K  V  T  K  P  L  P  D  L  G  F  E  L  L  T  K  I  S  F  L   75

SLS1_WT    tctgtggttacggacgttctgattgcattcctctcttcactgagcttctttaccttgtggaaactgtaccttctt   300
SLS1_R     AGCgtTgtGacAgaTgtGTtAatCgcGttTTtGAGCAGTTtATCTttTttCacACtCtggaaGTtAtaTTtGTtG
            S  V  V  T  D  V  L  I  A  F  L  S  S  L  S  F  F  T  L  W  K  L  Y  L  L   100

SLS1_WT    caccggcattgtgtggggtcgggtgaaccagaactcccatgtaacattcccggtgtgagtcgcttctttttatct   375
SLS1_R     caTAgAcaCtgCgtTggAAGCggCgaGccGgaGTtGccGtgCaaTatCccAggCgtTTCAAgAttTttCCtCAGC
            H  R  H  C  V  G  S  G  E  P  E  L  P  C  N  I  P  G  V  S  R  F  F  L  S   125

SLS1_WT    gtgtggttatgtaaggagaattgccgtattgaactgcgcaacgttcataccattgcatggatccgttttattaca   450
SLS1_R     gtTtggCtCtgCaaAgaAaaCtgTcgCatCgaGTtAcgTaaTgtGcaCacTatCgcGtggatTcgCttCatCacG
            V  W  L  C  K  E  N  C  R  I  E  L  R  N  V  H  T  I  A  W  I  R  F  I  T   150

SLS1_WT    tcatacgcactcctgctactctttcgctcgctggttattgttatgacgtccatgccgacgccagtggacaagtgc   525
SLS1_R     AGCtaTgcGTtActCTTGTTAttCcgTAGCctCgtGatCgtGatgacAAGTatgccAacAccGgtTgaTaaAtgT
            S  Y  A  L  L  L  L  F  R  S  L  V  I  V  M  T  S  M  P  T  P  V  D  K  C   175

SLS1_WT    caaaacccgccgaaaatagagaaccccgtaaagaacgttatactcactgttcttacagctggtggtggttccata   600
SLS1_R     caGaaTccAccAaaGatCgaAaaTccTgtCaaAaaTgtGatCTtAacCgtGTtGacGgcCggCggCggCAGTatC
            Q  N  P  P  K  I  E  N  P  V  K  N  V  I  L  T  V  L  T  A  G  G  G  S  I   200

SLS1_WT    cactgtggtgatctcatgtacagtggccatactgtgattctgacgcttcatctcatgttccactggatttatggg   675
SLS1_R     caTtgCggCgaCTtGatgtaTTCCggTcaCacCgtTatCctCacATtGcaCTtGatgttTcaTtggatCtaCggA
            H  C  G  D  L  M  Y  S  G  H  T  V  I  L  T  L  H  L  M  F  H  W  I  Y  G   225

SLS1_WT    gcaatggtacattggtcgtttcgtcctgtggtgactgtggtagcaatttttggctactattgcatcgttgcctct   750
SLS1_R     gcGatggtCcaCtggAGCttCAgGccCgtTgtTacCgtTgtCgcGatCttCggTtaTtaCtgTatTgtGgcTAGt
            A  M  V  H  W  S  F  R  P  V  V  T  V  V  A  I  F  G  Y  Y  C  I  V  A  S   250
                                                                                (I)
SLS1_WT    cgctcccattacacagatgacgtgttggtagctatttatttaacaattgcaacattcatagcagtaggtcacaac   825
SLS1_R     cgTAGTcaCtaTacGgaCgaTgtTCtCgtCgcCatCtaCCtCacGatCgcGacGttTatCgcGgtCggCcaTaaT
            R  S  H  Y  T  D  D  V  L  V  A  I  Y  L  T  I  A  T  F  I  A  V  G  H  N   275
                                                                     (II)
SLS1_WT    gctgatggagctccatggcagctgcaactttttattcgctggttgccatgttgtggtgccaattcacgtgaagtg   900
SLS1_R     gcGgaCggagctccatggcagctgcaactttttattcgctggttgccatgttgtggtgccaattcacgtgaagtg
            A  D  G  A  P  W  Q  L  Q  L  F  I  R  W  L  P  C  C  G  A  N  S  R  E  V   300
                                                                           (III)
SLS1_WT    acggaagacagccaaccggtaatggtggcttttaagagtgaagcagtggatgagttacgagagagggacgatagt   975
SLS1_R     acggaagacagccaaccggtaatggtggcttttaagagtgaagcagtggatgagttacgagagagggacgatagt
            T  E  D  S  Q  P  V  M  V  A  F  K  S  E  A  V  D  E  L  R  E  R  D  D  S   325
                                                          XmaI
SLS1_WT    gcggggttgagttgtgaggtatcaactaacgaggtatgacccggg                                  1014
SLS1_R     gcggggttgagttgtgaggtatcaactaacgaggtatgacccggg
            A  G  L  S  C  E  V  S  T  N  E  V  -                                         337
```

Fig S2B. TbSLS2 (EPC synthase) (Tb927.9.9400)

```
                                                              EcoRI
                                                             gaattcATGtacccttacgatgtg
                                                                    M  Y  P  Y  D  V

                         3xHA EPITOPE                                          NheI
          cctgattacgcgtacccatacgacgtgccagactacgcatacccgtacgatgtgcccgattacgcagggggctagc
           P  D  Y  A  Y  P  Y  D  V  P  D  Y  A  Y  P  Y  D  V  P  D  Y  A  G  A  S

SLS2_WT   atggctgtcccaccagtggaaatgtatagtggttccttttggaacagaatgaggaaacccttgccattacgaacg   75
SLS2_R    atggcGgtTccGccGgtTgaGatgtaCTCAggCAGTttCtggaaTCgGatgCgAaaGccACtCccGCtCAgGacA
           M  A  V  P  P  V  E  M  Y  S  G  S  F  W  N  R  M  R  K  P L  P  L  R  T     25

SLS2_WT   caagtcattcgattcacagttgtattcgtaattgttgtctttatccttgctgttttgttgcaaatcacacatgaa   150
SLS2_R    caGgtAatCAgGttTacCgtGgtCttTgtCatCgtCgtAttCatATtGgcGgtGCtCCtCcaGatAacCcaCgaG
           Q  V  I  R  F  T  V  V  F  V  I  V  V  F  I  L  A  V  L  L  Q  I  T  H  E    50

SLS2_WT   cgtatgccggaccccaaggtgacgaagccgctaccagatctcggttttgaggtattgcacaagtatcctttttg   225
SLS2_R    AgGatgccAgaTccAaaAgtTacAaaAccATtGccGgaCTtAggCttCgaAgtCCtCcaTaaAtaCccCttCCtC
           R  M  P  D  P  K  V  T  K  P  L  P  D  L  G  F  E  V  L  H  K  Y  P  F  L    75

SLS2_WT   ttttctgtcgcagactgctgtatcggtttcttgaatattctgagtgttttcaccgctttcaaactgtaccttctt   300
SLS2_R    ttCAGCgtAgcGgaTtgTtgCatAggCttTCtTaaCatCctCTCCgtGttTacTgcCttTaaGctCtaTTtGTtG
           F  S  V  A  D  C  C  I  G  F  L  N  I  L  S  V  F  T  A  F  K  L  Y  L  L    100

SLS2_WT   caccggcattgtgtggggtcgggtgaaccagaactcccatgtaacattcccggtgtgagtcgcttctttttatct   375
SLS2_R    caTAgAcaCtgCgtTggAAGCggCgaGccGgaGTtGccGtgCaaTatCccAggCgtTTCAAgAttTttCCtCAGC
           H  R  H  C  V  G  S  G  E  P  E  L  P  C  N  I  P  G  V  S  R  F  F  L  S    125

SLS2_WT   gtgtggttatgtaaggagaattgccgtattgaactgcgcaacgttcataccattgcatggatccgttttattaca   450
SLS2_R    gtTtggCtCtgCaaAgaAaaCtgTcgCatCgaGTtAcgTaaTgtGcaCacTatCgcGtggatTcgCttCatCacG
           V  W  L  C  K  E  N  C  R  I  E  L  R  N  V  H  T  I  A  W  I  R  F  I  T    150

SLS2_WT   tcatacgcactcctgctactctcccgttcagttattatggtagtgacgtcgcttcccaatcccgatgacctgtgc   525
SLS2_R    AGCtaTgcGTtActCTtGTtAAGTcgCAGCgtGatCatggtCgtTacAAGCTtGccTaaCccTgaCgaTTtAtgT
           S  Y  A  L  L  L  L  S  R  S  V  I  M  V  V  T  S  L  P  N  P  D  D  L  C    175

SLS2_WT   caagacccgccgaaaatagaaaatcgtgtgaaagatgtcatacttactgttcttacagctggtgccggttccata   600
SLS2_R    caGgaTccAccAaaGatCgaGaaCcgCgtTaaGgaCgtAatCTtGacCgtGTtGacGgcCggCgcTggCAGTatC
           Q  D  P  P  K  I  E  N  R  V  K  D  V  I  L  T  V  L  T  A  G  A  G  S  I    200

SLS2_WT   cactgtggtgatctcatgtacagtggccatactgtgattctgacgcttcatctcatgttccactggatttatggg   675
SLS2_R    caTtgCggCgaCTtGatgtaTTCAggTcaCacCgtTatCTtAacATtGcaCTtGatgttTcaTtggatCtaCggA
           H  C  G  D  L  M  Y  S  G  H  T  V  I  L  T  L  H  L  M  F  H  W  I  Y  G    225

SLS2_WT   gcaatggtacattggtcgtttcgtcctgtggtgactgtggtagcaattttggctactattgcatcgttgcctct   750
SLS2_R    gcGatggtCcaCtggAGCttCAgGccCgtTgtTacCgtTgtCgcGatCttCggTtaTtaCtgTatTgtGgcTAGt
           A  M  V  H  W  S  F  R  P  V  V  T  V  V  A  I  F  G  Y  Y  C  I  V  A  S    250
                                                                         (I)
SLS2_WT   cggttccattacacagatgacgtgttggtagctatttatttaacaattgcaacattcatagcagtaggtcacaac   825
SLS2_R    AgAttTcaCtaTacGgaCgaTgtTCtCgtCgcCatCtaCCtCacGatCgcGacGttTatCgcGgtCggCcaTaaT
           R  F  H  Y  T  D  D  V  L  V  A  I  Y  L  T  I  A  T  F  I  A  V  G  H  N    275
                                                             (II)
SLS2_WT   gctgatggagctccatggcagctgcaacttttttattcgctggttgccatgttgtggtgccaattcacgtgaagtg   900
SLS2_R    gcGgaCggagctccatggcagctgcaacttttttattcgctggttgccatgttgtggtgccaattcacgtgaagtg
           A  D  G  A  P  W  Q  L  Q  L  F  I  R  W  L  P  C  C  G  A  N  S  R  E  V    300
                                                           (III)                XmaI
SLS2_WT   actgaagatggcgtacctgttgcaatcgtaattaaaaacgaagaaatgatgaatttcgagggaaagtcatagcccggg 972
SLS2_R    actgaagatggcgtacctgttgcaatcgtaattaaaaacgaagaaatgatgaatttcgagggaaagtcatagcccggg
           T  E  D  G  V  P  V  A  I  V  I  K  N  E  E  M  M  N  F  E  G  K  S  -       323
```

Fig S2C. TbSLS3 (SM/EPC synthase) (Tb927.9.9390)

```
                                                EcoRI          HA EPITOPE        NheI
                                               gaattcATGtacccatacgacgtcccagattacgcggctagc
                                                      M  Y  P  Y  D  V  P  D  Y  A  A  S

SLS3_WT   atggctgtcccaccagtggaaatgtatagtggttccttttggaacagaatgaggaaacccttgccattacgaacg    75
SLS3_R    atggcGgtTccGccGgtTgaGatgtaCTCAggCAGTttCtggaaTCgGatgCgAaaGccACtCccGCtCAgGacA
           M  A  V  P  P  V  E  M  Y  S  G  S  F  W  N  R  M  R  K  P  L  P  L  R  T    25

SLS3_WT   caagtcattcgattcacagttgtattcgtaatcgtctctttcatccttgcggttgcgttgcaaatcacacatgaa   150
SLS3_R    caGgtAatCAgGttTacCgtGgtCttTgtCatAgtAAGCttTatATtGgcAgtGgcACtCcaGatAacCcaCgaG
           Q  V  I  R  F  T  V  V  F  V  I  V  S  F  I  L  A  V  A  L  Q  I  T  H  E    50

SLS3_WT   cgtatgccggaccccaaggtgacgaagccgctaccagatctcggttttgaacttttgacgaaggtaccaggtatg   225
SLS3_R    AgGatgccAgaTccAaaAgtTacAaaAccATtGccGgaCTtAggCttCgaGTtGCtCacAaaAgtGccGggCatg
           R  M  P  D  P  K  V  T  K  P  L  P  D  L  G  F  E  L  L  T  K  V  P  G  M    75

SLS3_WT   tatgttcttgcagactgctgtatcggtttcttgaatattctgagtgttttcaccgctttcaaactgtaccttctt   300
SLS3_R    taCgtGTtGgcTgaTtgTtgCatAggCttTCtTaaCatCTtATCCgtGttTacTgcCttTaaGTtAtaTTtGTtG
           Y  V  L  A  D  C  C  I  G  F  L  N  I  L  S  V  F  T  A  F  K  L  Y  L  L   100

SLS3_WT   caccggcattgtgtggggtcgggtgaaccagaactcccatgtaacattcccggtgtgagtcgcttctttttatct   375
SLS3_R    caTAgAcaCtgCgtTggAAGCggCgaGccGgaGTtAccGtgCaaTatCccTggCgtTTCAAgAttTttCCtCAGC
           H  R  H  C  V  G  S  G  E  P  E  L  P  C  N  I  P  G  V  S  R  F  F  L  S   125

SLS3_WT   gtgtggttatgtaaggagaattgccgtattgaactgcgcaacgttcataccattgcatggatccgttttattaca   450
SLS3_R    gtTtggCtCtgCaaAgaAaaCtgTAgGatCgaGTtAAgAaaTgtGcaCacTatCgcGtggatAAgGttCatCacG
           V  W  L  C  K  E  N  C  R  I  E  L  R  N  V  H  T  I  A  W  I  R  F  I  T   150

SLS3_WT   tcatacgcactcctgctgctctttcgctcggtggttattgtcatgacatcctttcctgctcccgatgacctgtgc   525
SLS3_R    AGCtaTgcGTtGTtATtATtGttCAgGAGCgtAgtGatCgtAatgacGAGCttCccCgcCccTgaCgaTTtAtgT
           S  Y  A  L  L  L  L  F  R  S  V  V  I  V  M  T  S  F  P  A  P  D  D  L  C   175

SLS3_WT   caaaacccgccgaaaatagagaaccccgtaaagaacgttatacttactgttcttacagctggtggtggttccata   600
SLS3_R    caGaaTccAccAaaGatCgaAaaTccAgtCaaAaaTgtGatCTtGacCgtGTtGacGgcCggCggCggCAGTatC
           Q  N  P  P  K  I  E  N  P  V  K  N  V  I  L  T  V  L  T  A  G  G  G  S  I   200

SLS3_WT   cactgtggtgatctcatgtacagtggccatactgtgattctgacgcttcatctcatgttccactggatttatggg   675
SLS3_R    caTtgCggCgaCTtGatgtaTTCAggTcaCacCgtAatCTtAacATtGcaCTtGatgttTcaTtggatCtaCggA
           H  C  G  D  L  M  Y  S  G  H  T  V  I  L  T  L  H  L  M  F  H  W  I  Y  G   225

SLS3_WT   gcaatggtacattggtcgtttcgtcctgtggtgactgtggtagcaatttttagctactattgcatcgttgcctct   750
SLS3_R    gcGatggtCcaCtggAGCttCAgGccCgtTgtTacCgtTgtCgcTatCttCTCGtaTtaCtgTatTgtGgcTAGt
           A  M  V  H  W  S  F  R  P  V  V  T  V  V  A  I  F  S  Y  Y  C  I  V  A  S   250
                                                                     (I)
SLS3_WT   cggttccattacacagatgacgtgttggtagctatttatttaacaattgcaacattcatagcagtaggtcacaac   825
SLS3_R    AgAttTcaCtaTacGgaCgaTgtTCtCgtCgcAatCtaCCtCacGatCgcGacGttTatCgcGgtCggCcaTaaT
           R  F  H  Y  T  D  D  V  L  V  A  I  Y  L  T  I  A  T  F  I  A  V  G  H  N   275
                                                    (II)
SLS3_WT   gctgatggagctccatggcagctgcaacttttattcgctggtggccatgttgtggtgccaattcacgtgaaatg   900
SLS3_R    gcGgaCggagctccatggcagctgcaacttttattcgctggtggccatgttgtggtgccaattcacgtgaaatg
           A  D  G  A  P  W  Q  L  Q  L  F  I  R  W  W  P  C  C  G  A  N  S  R  E  M   300
                                                                  (III)
SLS3_WT   acggaagacagccaaccggtaatggtggcttttaagagtgaagcggcgggtcagtctagcaggaaagtcgtagat   975
SLS3_R    acggaagacagccaaccggtaatggtggcttttaagagtgaagcggcgggtcagtctagcaggaaagtcgtagat
           T  E  D  S  Q  P  V  M  V  A  F  K  S  E  A  A  G  Q  S  S  R  K  V  V  D   325
                     XmaI
SLS3_WT   gagagaaatcattgacccggg                                                         990
SLS3_R    gagagaaatcattgacccggg
           E  R  N  H  -                                                                329
```

Fig S2D. TbSLS4 (SM/EPC synthase) (Tb927.9.9380)

```
                                        EcoRI          HA EPITOPE            NheI
                                        gaattcATGtacccatacgacgtcccagattacgcggctagc
                                                 M  Y  P  Y  D  V  P  D  Y  A  A  S

SLS4_WT   atggctgtcccaccagtggaaatgtatagtggttcctttggaacagaatgaggaaacccttgccattacgaacg     75
SLS4_R    atggcGgtGccGccGgtggaGatgtaCTCAggCAGTttCtggaaTCgGatgCgGaaGccTCtCccGCtCAgGacA
           M  A  V  P  P  V  E  M  Y  S  G  S  F  W  N  R  M  R  K  P  L  P  L  R  T     25

SLS4_WT   caagtcattcgattcacagttgtattcgtaatcgtctctttcatccttgcggttgcgttgcaaatcacacatgaa    150
SLS4_R    caGgtAatCAgGttTacGgtGgtCttTgtCatTgtAAGCttTatTTtGgcAgtGgcACtCcaGatAacGcaCgaG
           Q  V  I  R  F  T  V  V  F  V  I  V  S  F  I  L  A  V  A  L  Q  I  T  H  E     50

SLS4_WT   cgtatgccggaccccaaggtgacgaagccgctaccagatctcggttttgaacttttgacgaaggtaccaggtatg    225
SLS4_R    AgGatgccAgaTccTaaAgtTacAaaAccATtGccGgaCTTGggCttCgaGTtGCtTacAaaAgtGccGggCatg
           R  M  P  D  P  K  V  T  K  P  L  P  D  L  G  F  E  L  L  T  K  V  P  G  M     75

SLS4_WT   tatgttcttgcagactgctgtatcggtttcttgaatattctgagtgttttcaccgctttcaaactgtaccttctt    300
SLS4_R    taCgtGTtGgcTgaTtgTtgCatAggCttTCtTaaCatCctCTCCgtGttTacTgcCttTaaGctCtaTTtGTtG
           Y  V  L  A  D  C  C  I  G  F  L  N  I  L  S  V  F  T  A  F  K  L  Y  L  L    100

SLS4_WT   caccggcattgtgtggggtcgggtgaaccagaactcccatgtaacattcccggtgtgagtcgcttctttttatct    375
SLS4_R    caTAgAcaCtgCgtTggAAGCggCgaGccGgaGTtAccGtgCaaTatCccTggCgtTTCAAgAttTttCCtCAGC
           H  R  H  C  V  G  S  G  E  P  E  L  P  C  N  I  P  G  V  S  R  F  F  L  S    125

SLS4_WT   gtgtggttatgtaaggagaattgccgtattgaactgcgcaacgttcataccattgcatggatccgttttattaca    450
SLS4_R    gtTtggCtCtgCaaAgaAaaCtgTAgGatCgaGctCAgAaaTgtGcaCacTatCgcGtggatAAgGttCatCacG
           V  W  L  C  K  E  N  C  R  I  E  L  R  N  V  H  T  I  A  W  I  R  F  I  T    150

SLS4_WT   tcatacgcactcctgctactctttcgctcggtagttattgtcatgacatcgcttcctgctcccgatgacctgtgc    525
SLS4_R    AGCtaTgcGTtGctCTtGTtGttCAgGAGCgtCgtGatCgtAatgacGAGCTtGccCgcCccTgaCgaTTtAtgT
           S  Y  A  L  L  L  L  F  R  S  V  V  I  V  M  T  S  L  P  A  P  D  D  L  C    175

SLS4_WT   caagacccgccgaaaatagagaaccccgtaaagaacgttatacttactgttcttacagctggtggtggttccata    600
SLS4_R    caGgaTccAccAaaGatCgaAaaTccAgtCaaAaaTgtGatCTtGacCgtGTtGacGgcCggCggCggCAGTatC
           Q  D  P  P  K  I  E  N  P  V  K  N  V  I  L  T  V  L  T  A  G  G  G  S  I    200

SLS4_WT   cactgtggtgatctcatgtacagtggccatactgtgattctgacgcttcatctcatgttccactggatttatggg    675
SLS4_R    caTtgCggCgaCTtGatgtaTTCAggTcaCacCgtTatCTtAacATtGcaCTtGatgttTcaTtggatCtaCggA
           H  C  G  D  L  M  Y  S  G  H  T  V  I  L  T  L  H  L  M  F  H  W  I  Y  G    225

SLS4_WT   gcaatggtacattggtcgtttcgtcctgtggtgactgtggtagcaattttggctactattgcatcgttgcctct    750
SLS4_R    gcGatggtCcaCtggAGCttCcgCccCgtTgtTacCgtTgtCgcTatCttCggTtaTtaCtgTatTgtGgcTAGt
           A  M  V  H  W  S  F  R  P  V  V  T  V  V  A  I  F  G  Y  Y  C  I  V  A  S    250
                                                                          (I)
SLS4_WT   cggttccattacacagatgacgtgttggtagctatttatttaacaattgcaacattcatagcagtaggtcacaac    825
SLS4_R    AgAttTcaCtaTacCgaCgaTgtTCtAgtCgcCatCtaCCtCacGatCgcGacGttTatCgcGgtCggCcaTaaT
           R  F  H  Y  T  D  D  V  L  V  A  I  Y  L  T  I  A  T  F  I  A  V  G  H  N    275
                                                                (II)
SLS4_WT   gctgatggagctccatggcagctgcaacttttattcgctggttgccatgttgtggtgccaattcacgtgaaatg    900
SLS4_R    gcCgaCggagctccatggcagctgcaacttttattcgctggttgccatgttgtggtgccaattcacgtgaaatg
           A  D  G  A  P  W  Q  L  Q  L  F  I  R  W  L  P  C  C  G  A  N  S  R  E  M    300
                                                                   (III)
SLS4_WT   acggaagatagccaaccggtaatggtggcttttaagagtgaagaattagatgaaatgaatggtgtgctggagggc    975
SLS4_R    acggaagatagccaaccggtaatggtggcttttaagagtgaagaattagatgaaatgaatggtgtgctggagggc
           T  E  D  S  Q  P  V  M  V  A  F  K  S  E  E  L  D  E  M  N  G  V  L  E  G    325
                                                                              XmaI
SLS4_WT   agacaaaaaaaacatggtggtgttggtgatggtgaagctttaatgtttaaatgtggggcgtatgtgtgacccggg   1044
SLS4_R    agacaaaaaaaacatggtggtgttggtgatggtgaagctttaatgtttaaatgtggggcgtatgtgtgacccggg
           R  Q  K  K  H  G  G  V  G  D  G  E  A  L  M  F  K  C  G  A  Y  V  -          340
```

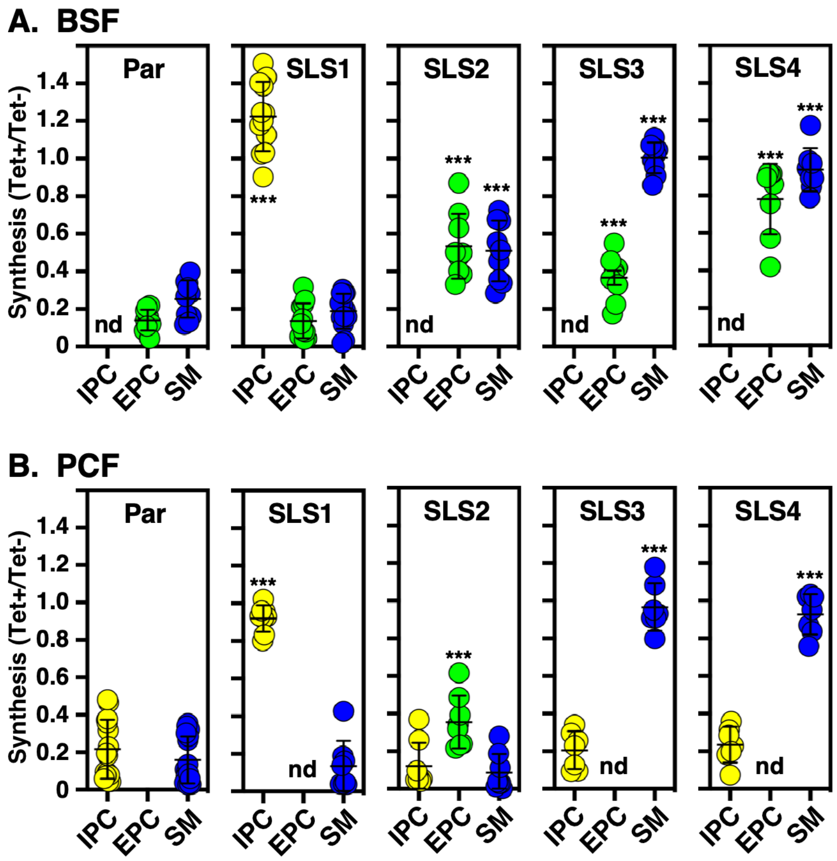

Figure S3. Stage-Specific Sphingolipid Synthesis. Quantitative data from Figs 4C & 8C are presented for direct comparison.

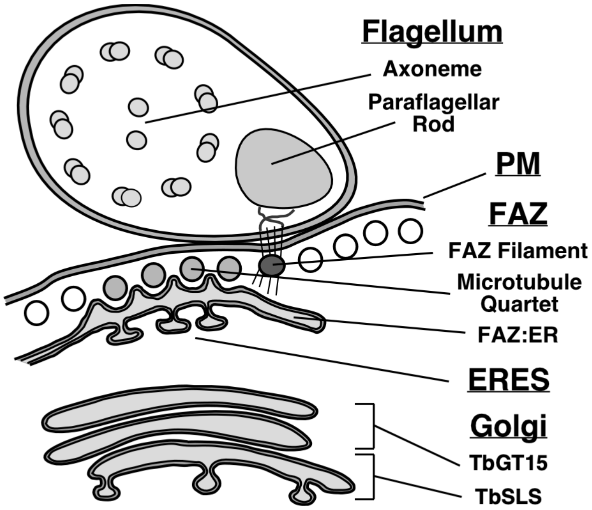

Figure S4. Diagram of FAZ:ERES:Golgi junctional region. A cross sectional diagram through an ERES:Golgi junction in the region where the flagellum associates with the plasma membrane (PM) and underlying flagellar adherence zone (FAZ). Internal organelles are the FAZ-associated ER (FAZ:ER), ER exit sites (ERES) and Golgi. Substructures are labeled as appropriate. The relative location of TbGT15 and TbSLSs is from images presented in Figs. 4 and 8. Adapted from Sevova & Bangs (2009) *Mol. Biol. Cell*, 20:4739.
